# Covalently Conjugated NOD2/TLR7 Agonists Are Potent and Versatile Immune Potentiators

**DOI:** 10.1021/acs.jmedchem.2c00808

**Published:** 2022-11-06

**Authors:** Samo Guzelj, Matjaž Weiss, Bram Slütter, Ruža Frkanec, Žiga Jakopin

**Affiliations:** † Faculty of Pharmacy, University of Ljubljana, SI-1000 Ljubljana, Slovenia; ‡ Division of BioTherapeutics, Leiden Academic Centre for Drug Research, Leiden University, 2333 CC Leiden, The Netherlands; § Centre for Research and Knowledge Transfer in Biotechnology, University of Zagreb, 10000 Zagreb, Croatia

## Abstract

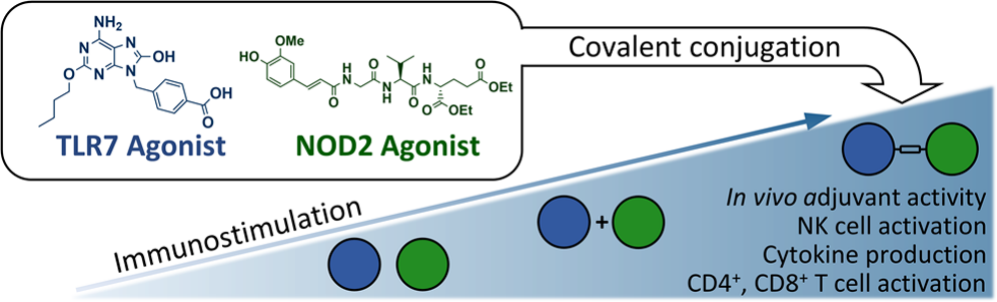

The success of vaccination with subunit vaccines often relies on the careful choice of adjuvants. There is great interest in developing new adjuvants that can elicit a cellular immune response. Here, we address this challenge by taking advantage of the synergistic cross-talk between two pattern recognition receptors: nucleotide-binding oligomerization-domain-containing protein 2 (NOD2) and Toll-like receptor 7 (TLR7). We designed two conjugated NOD2/TLR7 agonists, which showed potent immunostimulatory activities in human primary peripheral blood mononuclear cells and murine bone-marrow-derived dendritic cells. One of these, **4**, also generated a strong antigen-specific immune response *in vivo*, with a Th1-polarized profile. Importantly, our study shows that novel NOD2/TLR7 agonists elicit sophisticated and fine-tuned immune responses that are inaccessible to individual NOD2 and TLR7 agonists.

## Introduction

Vaccines have radically reduced morbidity and the number of infections and have even eliminated once devastating diseases. Although vaccines that incorporate recombinant antigens benefit from improved safety profiles compared to live attenuated vaccines, their weak immunogenicity requires the addition of vaccine adjuvants into the formulation. These act as immunopotentiators, to induce stronger and more durable immune responses to the co-administered antigen.^1^ Pattern recognition receptors (PRRs) detect and respond to distinct and well-defined pathogen-associated molecular patterns (PAMPs).^2^ By engaging PRRs, PAMPs control both the initial innate immune response and the subsequent formation of adaptive immunity, in terms of both intensity and shape. Therefore, synthetic PRR agonists have been at the forefront of vaccine adjuvant development.^3^

While any single PRR agonist can elicit an immune response, simultaneous activation of multiple PRRs can lead to synergistic signal amplification. In many ways, the coadministration of multiple PRR agonists imitates the immunogenicity of live attenuated vaccines, which carry multiple PAMPs and thus trigger complex sets of PRRs in a complementary manner.^4−6^ Given that these coordinated responses determine both the magnitude and the type of the provoked immune response, rational targeting of specific PRR combinations allows for the immune response to be tailored toward the most effective type of immunity required in protection against a particular pathogen. Moreover, several groups have demonstrated that covalent conjugation of multiple PRR agonists significantly enhances immunomodulatory activity compared to mixtures of unconjugated agonists.^7−15^ While adjuvants consisting of mixtures of unconjugated PRR agonists can rapidly diffuse through the immune system and may be more readily cleared, covalent conjugation ensures the delivery of all PRR-agonistic components to the same cell. Although indirect interactions between multiple cells through cytokines acting in a paracrine manner are possible, activation of multiple PRR signaling pathways and their corresponding cross-talk within the same cell provides stronger immune responses.^4,16^ The improved immune responses are also dose-sparing; i.e., they reduce the adjuvant and antigen dosing and improve the adjuvant safety profile.^4^

In this study, we present the development and immunostimulatory activity of conjugated agonists that take advantage of the synergistic cross-talk between nucleotide-binding oligomerization-domain-containing protein 2 (NOD2) and Toll-like receptor 7 (TLR7). NOD2 is a cytosolic receptor that is primarily involved in the detection of bacterial peptidoglycan fragments,^17,18^ while TLR7 is an endosomal receptor that is responsive to viral single-stranded RNA (ssRNA).^19^ Co-stimulation with NOD2 and TLR7 agonists has been reported to synergistically enhance cytokine production in peripheral blood mononuclear cells and macrophages.^20,21^ Furthermore, coengagement of both receptors has been shown to produce super-additive effects dendritic cell activation and development of humoral and cellular immune responses *in vivo*.^14,22−24^ Albeit exact mechanisms of NOD2/TLR7 cross-talk remain elusive, several possibilities have been proposed. For example, various combinations of NOD and TLR agonists have been reported to synergistically activate the downstream nuclear factor-κB (NF-κB) pathway. Given that many of the genes induced by NOD and TLR stimulation are regulated by NF-κB, this is indicative of interactions of signaling pathways directly downstream of NODs and TLRs.^16,25^ Besides NF-κB activation, other pathways could contribute to synergistic responses. For example, similar to TLR7, NOD2 has been reported to respond to viral ssRNA, leading to the activation of interferon regulatory factor-3 (IRF3) and production of type I interferons (IFNs).^26^ Indeed, a transcriptomic analysis of dendritic cells stimulated by agonists of both NOD2 and TLR7 has shown a prominent upregulation of interferon-stimulated genes (ISGs) in addition to the enhanced transcription of cytokines, chemokines, and costimulatory molecules.^23^ Furthermore, type I IFNs induced by several TLRs, including TLR7, have been shown to augment NOD2 expression, resulting in enhanced responsiveness to NOD2 agonists.^27^ In turn, NOD2 agonists have been reported to upregulate the expression of MyD88, an adaptor molecule in the TLR signaling pathway.^28,29^ While the involvement of these cross-regulatory mechanisms in the synergistic amplification of immune effector functions remains poorly understood, we show here that the cooperative signaling between NOD2 and TLR7 can be successfully harnessed with covalently conjugated NOD2/TLR7 agonists.

## Results and Discussion

**Figure 1:**
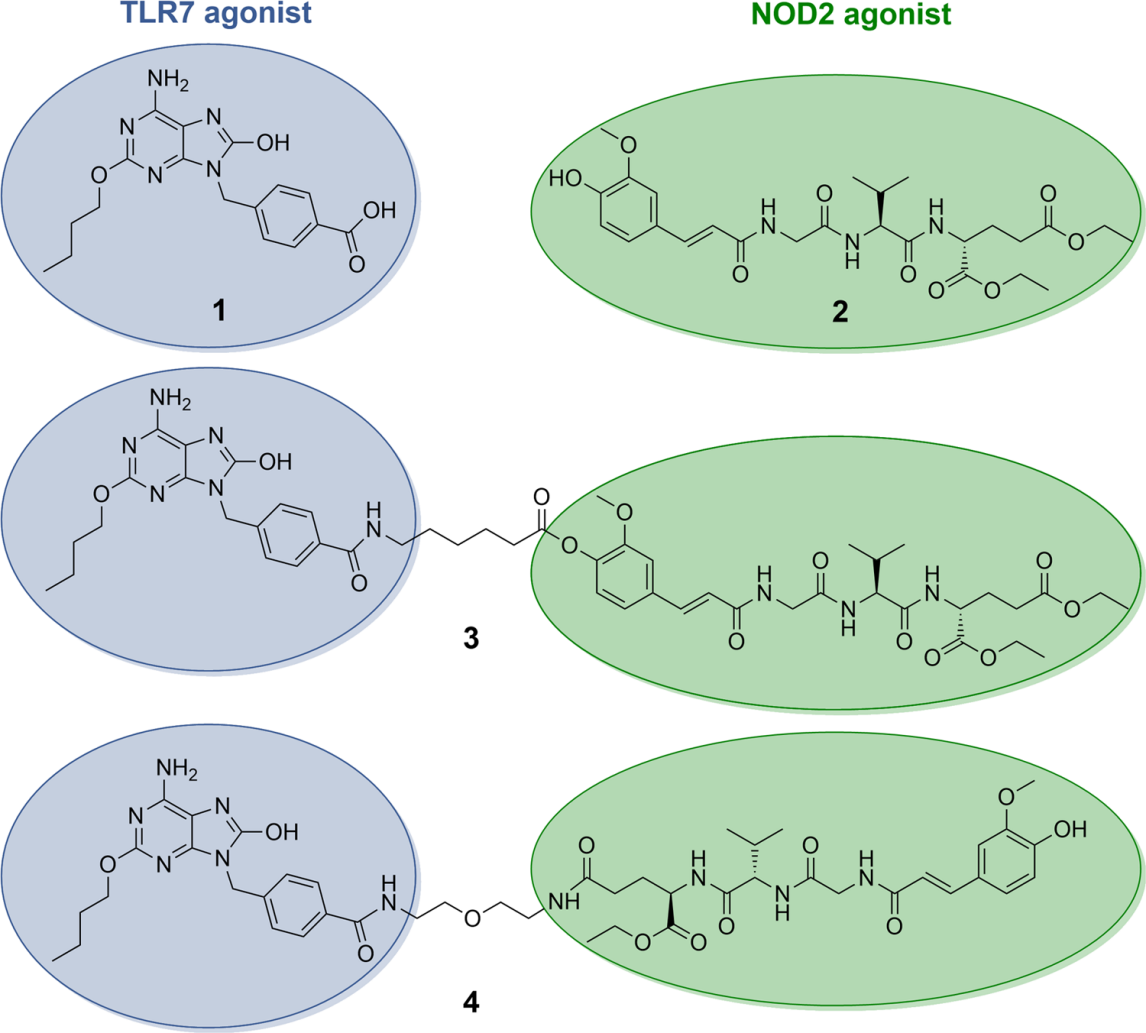
Chemical structures of the TLR7 agonist **1**, the NOD2 agonist **2**, and the conjugated NOD2/TLR7 agonists **3** and **4**.

The conjugated NOD2/TLR7 agonists **3** and **4** (Figure 1) were constructed by linking our flagship in-house desmuramylpeptide NOD2 agonist **2**(30) and a purine-based TLR7 agonist **1**.^31,32^ The TLR7 agonist was chosen for its potent TLR7 agonist activity as well as the presence of a carboxylic acid group, which has previously been shown to be a suitable site for conjugation.^32^ Accordingly, both conjugates were synthesized by first attaching a spacer molecule to this group. In **3**, the 6-aminohexanoic acid spacer is connected to the phenol group of the NOD2 agonist moiety via a cleavable ester functionality. Conversely, **4** incorporates a bis(2-aminoethyl)ether spacer attached to the ω-carboxylic acid of the d-glutamic acid moiety through a metabolically more stable amide bond.

**Scheme 1:**
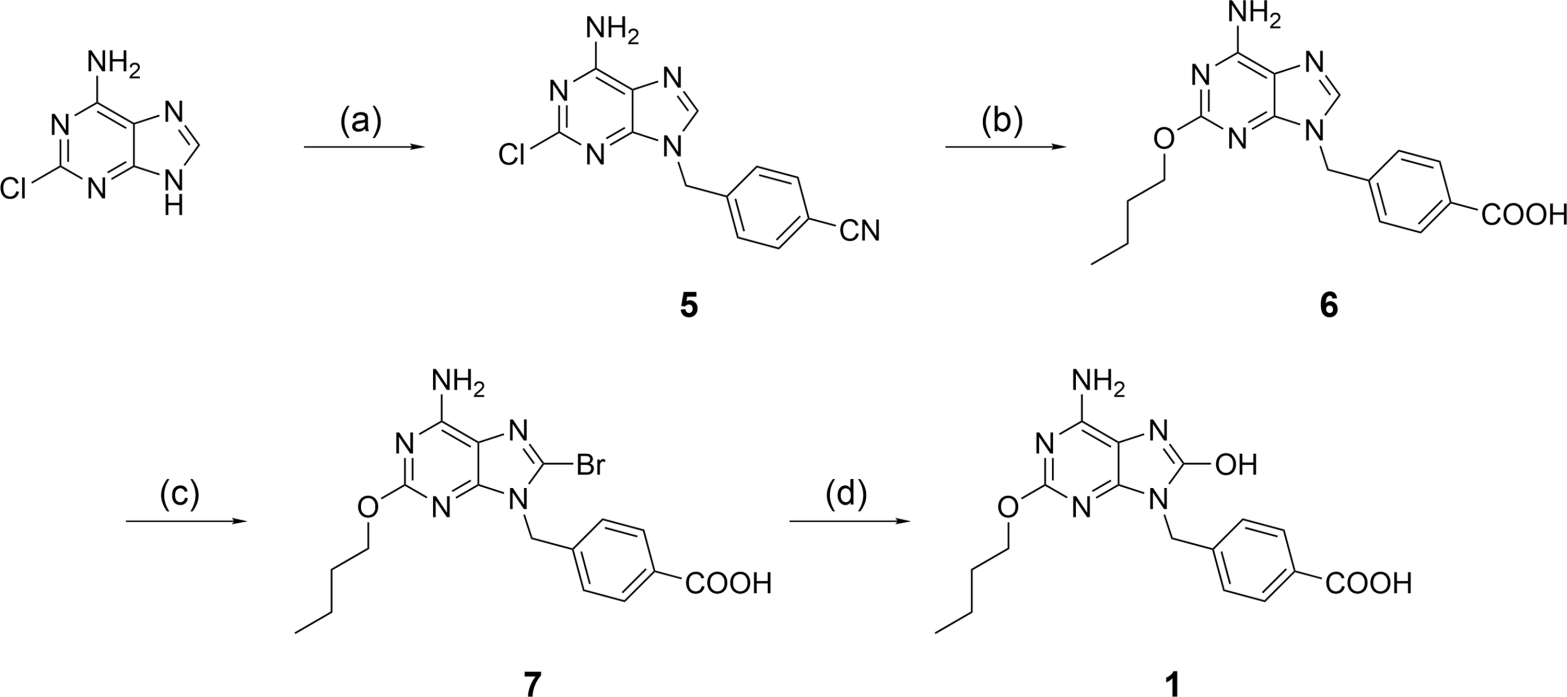
Synthesis of the TLR7 Building Block **1** Reagents and conditions: (a) 4-(bromomethyl)benzonitrile, K_2_CO_3_, DMSO, rt; (b) (i) *n*-BuONa, *n*-BuOH, reflux; (ii) H_2_O, reflux; (c) Br_2_, CH_3_COONa, CH_3_COOH, rt; (d) NaOH, H_2_O, MeOH, reflux.

Scheme 1 illustrates the four-step synthesis of the TLR7 agonist, which begins with the benzylation of commercially available 2-chloroadenine yielding compound **5**. Following the introduction of a butoxy substituent with sodium butoxide, water was added directly to the reaction mixture to initiate nitrile hydrolysis, giving the carboxylic acid intermediate **6**. Bromination of this intermediate with bromine and sodium acetate in acetic acid gave **7**, which was hydrolyzed with sodium hydroxide to afford the TLR7 agonist **1**.

**Scheme 2:**
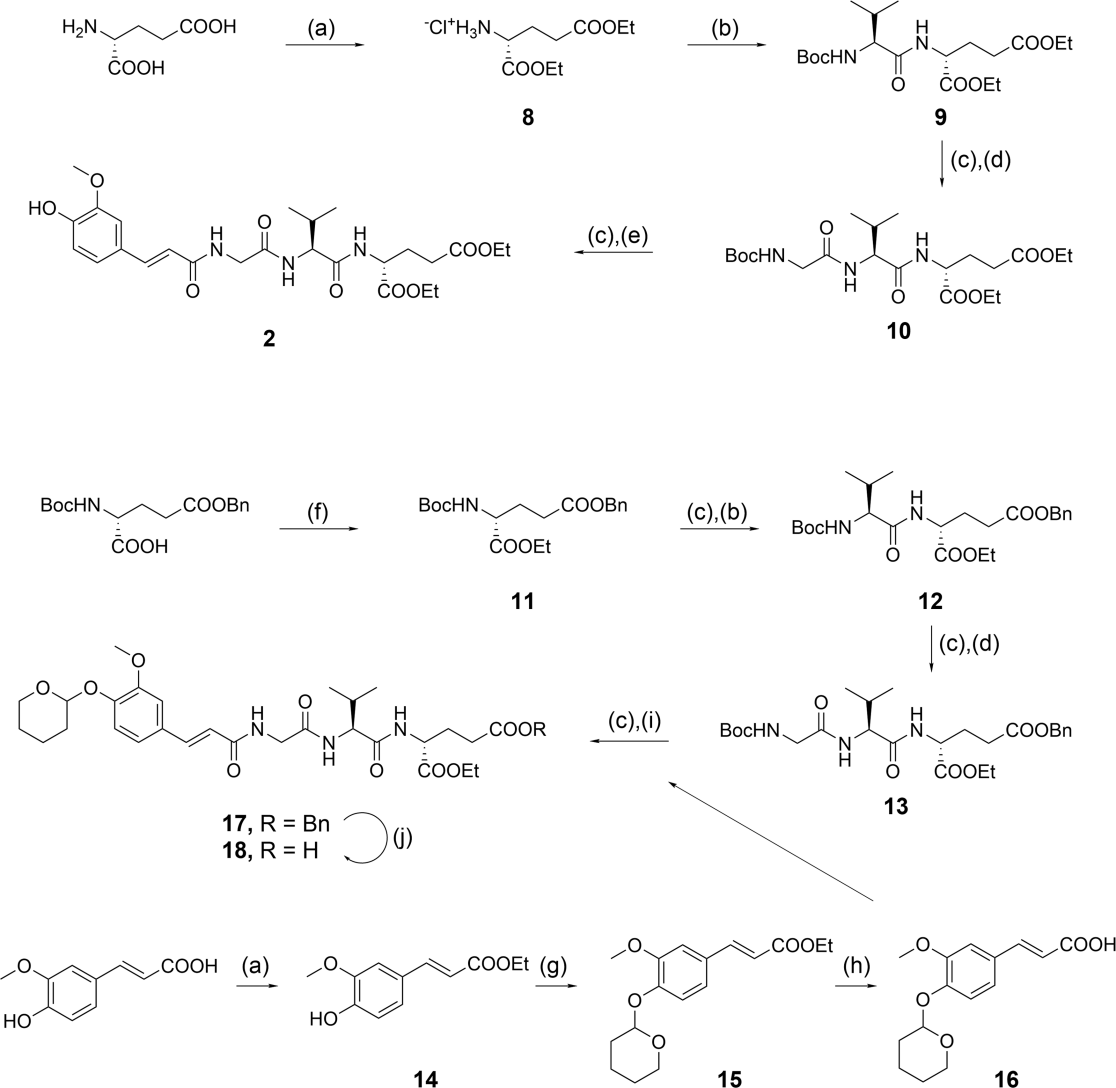
Synthesis of NOD2 Agonist Building Blocks **2** and **18** Reagents and conditions: (a) SOCl_2_, EtOH, reflux; (b) Boc-l-Val, EDC, HOBt, DIPEA, DMAP, DMF, rt; (c) TFA/DCM (1:5), rt; (d) Boc-Gly, EDC, HOBt, DIPEA, DMAP, DMF, rt; (e) *trans*-ferulic acid, EDC, HOBt, DIPEA, DMAP, DMF, rt; (f) EtOH, EDC, DMAP, DCM, rt; (g) 3,4-dihydropyran, pyridinium *p*-toluenesulfonate, reflux; (h) NaOH, H_2_O, MeOH, rt; (i) EDC, HOBt, DIPEA, DMAP, DMF, rt; (j) Pd(OAc)_2_, Et_3_SiH, Et_3_N, DCM, rt.

To synthesize the NOD2 agonist intermediates (Scheme 2), ethyl ester groups were first introduced to d-glutamic acid or *N*-Boc-5-benzyl-d-glutamic acid to generate **8** and **11**, respectively. The Boc protecting group of **11** was cleaved with trifluoroacetic acid (TFA) and the free amines of **8** and deprotected **11** were coupled to Boc-l-valine to give dipeptides **9** and **12**. These were subjected to another round of TFA-mediated Boc deprotection and coupling to Boc-glycine to afford the tripeptides **10** and **13**. The diethyl ester derivative **10** was then deprotected and coupled to *trans*-ferulic acid to produce the NOD2 agonist **2**. Conversely, the 5-benzyl-1-ethyl derivative **13** was coupled to *trans*-ferulic acid, incorporating a tetrahydropyranyl-protected phenol group (**16**). This derivative was synthesized by first introducing an ethyl ester group to the carboxylic acid of *trans-*ferulic acid (**14**), followed by tetrahydropyranyl protection of the phenol group using 3,4-dihydropyran and pyridinium *p*-toluenesulfonate (**15**).^33^ A final basic hydrolysis yielded the protected *trans*-ferulic acid **16**, which was coupled with the Boc-deprotected **13** to produce the acyl tripeptide **17**. Debenzylation of **17** with hydrogenation over palladium/carbon also resulted in the reduction of the *trans*-ferulic acid double bond. A milder debenzylation method using palladium acetate, triethylsilane, and triethylamine was therefore employed to produce the second NOD2 agonist intermediate **18**.^34^

**Scheme 3:**
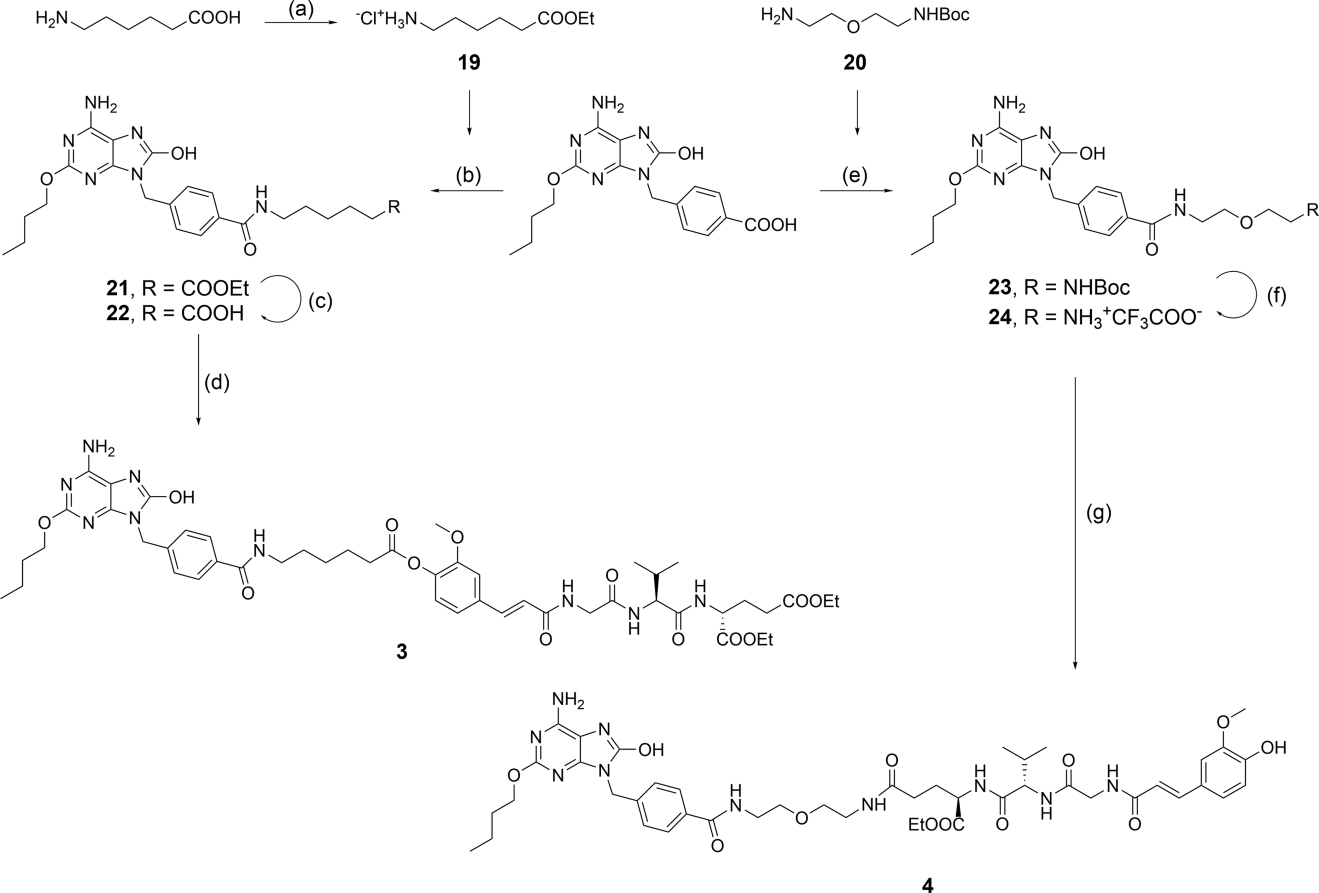
Final Assembly of NOD2/TLR7 Conjugates **3** and **4** Reagents and conditions: (a) SOCl_2_, EtOH, reflux; (b) **19**, COMU, Et_3_N, DCM, DMSO, rt; (c) NaOH, H_2_O, MeOH, rt; (d) **2**, COMU, DIPEA, DMF, rt; (e) **20**, COMU, DIPEA, DCM, DMSO, rt; (f) TFA/DCM (1:5), rt; (g) **18**, HATU, DIPEA, DMF, rt.

With the three agonist intermediates in hand, final assembly of the conjugates began by 1-[(1-(cyano-2-ethoxy-2-oxo-ethylideneaminooxy)-dimethylamino-morpholinomethylene)]methanaminium hexafluorophosphate (COMU)-mediated coupling with the mono-protected bifunctional linker molecules 6-aminohexanoic acid (**19**) and bis(2-aminoethyl)ether (**20**) to produce **21** and **23**, respectively (Scheme 3). Following hydrolytic deprotection of **21**, the resulting **22** was coupled to the NOD2 agonist intermediate **1** to generate the cleavable conjugate **3**. Similarly, Boc deprotection of **23** gave the free amine derivative **24**, which was coupled to the ω-carboxylic acid of the d-glutamic acid moiety of **18** to produce, after the acidolytic removal of the tetrahydropyranyl group during workup, the second conjugate **4**.

**Figure 2:**
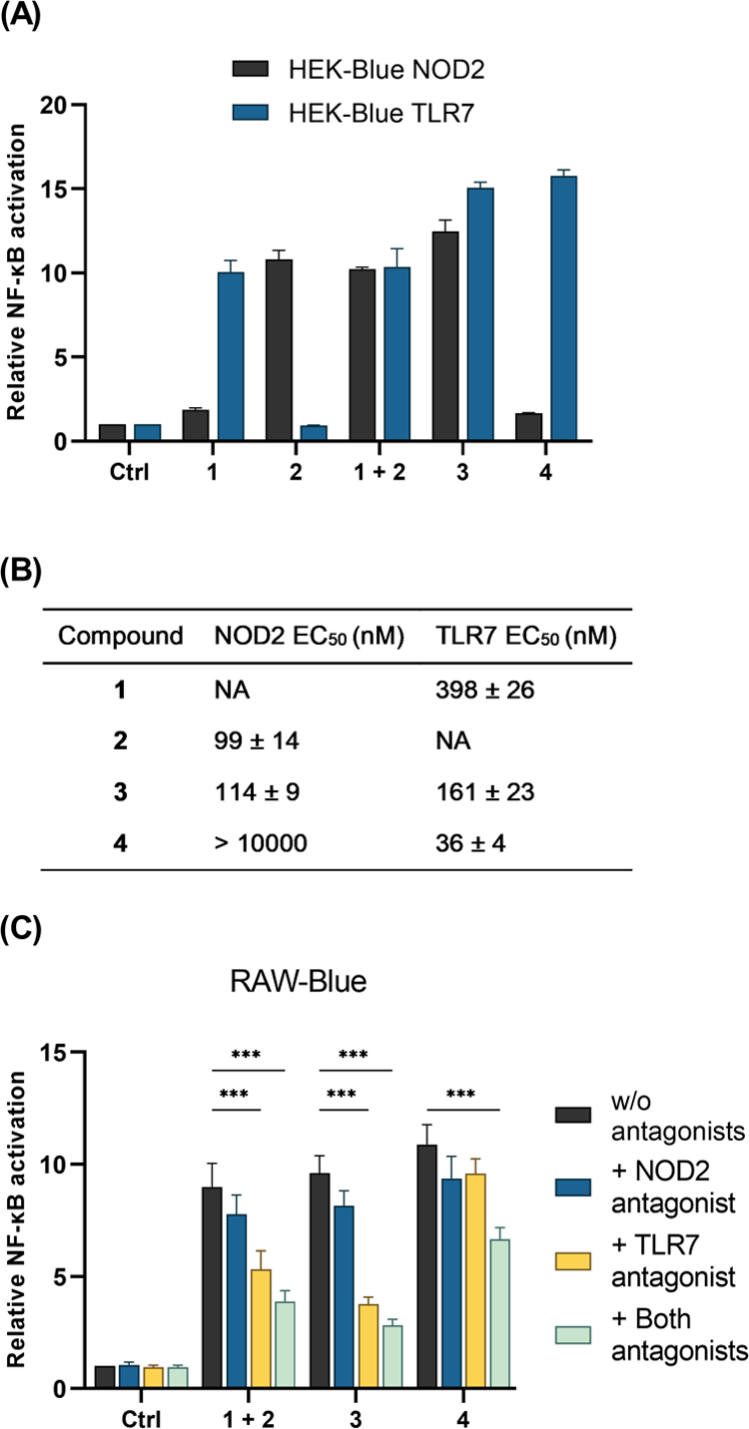
Receptor-specific NOD2 and TLR7 agonist activities of conjugated NOD2/TLR7 agonists. (A) HEK-Blue NOD2 or TLR7 cells were treated with the compounds (10 μM) for 18 h. The activities are shown relative to the vehicle-treated control (0.1% DMSO). Data are mean ± SEM of two (NOD2) or three (TLR7) independent experiments. (B) EC_50_ values determined in HEK-Blue NOD2 and TLR7 cells in at least three independent experiments with eight concentrations (1 nM to 10 μM); NA, not active. (C) RAW-Blue cells were treated with compounds (1 μM) for 18 h in the presence or absence of NOD2 antagonist **SG84** (5 μM), TLR7 antagonist **M5049** (1 μM), or their combination. The activities are shown relative to the vehicle-treated control (0.1% DMSO). Data are mean ± SEM of three independent experiments. ***, *p* < 0.001 *versus* relevant controls in the absence of antagonists (two-way ANOVA *post hoc* Dunnett’s test).

The conjugates were first evaluated for their receptor-specific NOD2 and TLR7 activities with commercially available HEK-Blue NOD2 and TLR7 reporter cell lines. After determining that none of these compounds showed cytotoxicity using the established MTS assay (Figure S1), activity tests showed that, as expected, both **1** and **2** selectively activated solely their cognate receptors (Figure 2A). Both conjugates activated TLR7; however, there was a marked difference in their EC_50_ values (Figure 2B). Compound **3** (EC_50_ = 161 nM) showed an approximately 2-fold improved TLR7 activity over **1** (EC_50_ = 398 nM), while **4** showed a 10-fold improved TLR7 activity (EC_50_ = 36 nM). Interestingly, only **3** (EC_50_ = 114 nM) activated NOD2, with roughly the same potency as **2** (EC_50_ = 99 nM). Compound **4**, on the other hand, activated NOD2 only to a minor extent at the highest concentration tested. In parallel, the conjugates were also evaluated using HEK-Blue NOD1 cells, which analogously to HEK-Blue NOD2 and TLR7 cells, express an NF-κB–inducible secreted embryonic alkaline phosphatase (SEAP) reporter gene. Neither conjugate activated HEK-Blue NOD1 cells at the highest concentration tested (10 μM), which confirmed that the results obtained with HEK-Blue NOD2 and TLR7 cells can be attributed exclusively to the activation of their respective receptors (Figure S2).

HEK293 cells possibly lack the enzymatic machinery needed for the cleavage of the amide bond between the spacer and the NOD2 agonist moiety in **4**. The ω-carboxylic acid of the d-glutamic acid moiety, which we previously showed to be essential for NOD2 binding,^30^ thus remains inaccessible. To test this hypothesis, we retested the compounds with the RAW-Blue cell line derived from the metabolically more active murine RAW264.7 macrophages, which also constitutively express both NOD2 and TLR7.^21^ Accordingly, both conjugates induced strong activation of RAW-Blue cells with comparable activities to that of the unconjugated mixture of agonists (Figures 2C and S2). To decouple the effects of individual moieties featured in conjugates, mechanistic studies were performed in the presence of a NOD2 antagonist,^35^ TLR7 antagonist,^36^ and their combination. The addition of a NOD2 or TLR7 antagonist reduced the activities of both conjugates as well as of the unconjugated mixture of both agonists (Figure 2C). The decrease in activity after antagonizing TLR7 was more prominent in the case of **3**, which is in line with its weaker TLR7 potency. The combination of both antagonists further decreased the conjugate-induced stimulation, which confirmed that the activities of the conjugates were reliant on both NOD2 and TLR7.

**Figure 3:**
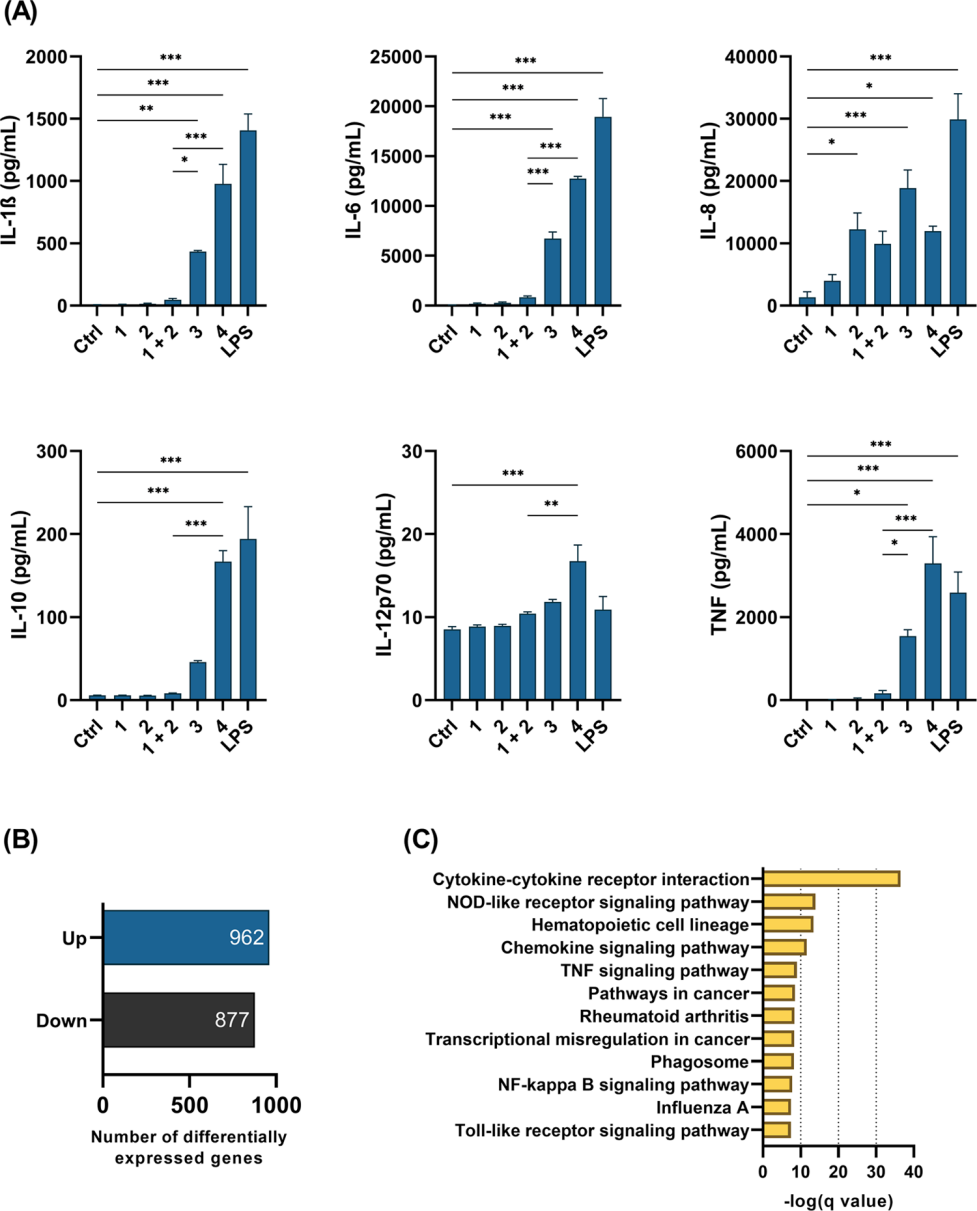
Immunostimulatory effects of conjugated NOD2/TLR7 agonists in human PBMCs. (A) Cytokine concentrations measured after 18 h stimulation with compounds (1 μM) or LPS (1 μg/mL). Data are mean ± SEM of four independent experiments. *, *p* < 0.05, **, *p* < 0.01, ***, *p* <0.001 (one-way ANOVA *post hoc* Bonferroni’s tests). (B) The number of significantly upregulated and downregulated genes in PBMCs from three independent donors after 18 h treatment with **4** (1 μM). A false discovery rate cutoff of <0.05 and a gene expression fold-change of >2 or <0.5 compared to the vehicle-treated controls (0.1% DMSO) were applied. (C) Top 12 most-enriched KEGG terms using the differentially expressed genes from (B) as the input for pathway enrichment analysis.

The immunostimulatory potential of the conjugates was then evaluated in human primary peripheral blood mononuclear cells (PBMCs). These represent a heterogeneous mixture of immune cell subpopulations, which enabled the study of concomitant NOD2/TLR7 activation in a physiologically more relevant system. Overnight stimulation of PBMCs with individual NOD2 and TLR7 agonists induced only minor increases in cytokine production, with only IL-8 showing any significant increase after stimulation with **2** (Figure 3A). Both conjugates, on the other hand, elicited extensive proinflammatory responses, as shown by the significant increases in the production of IL-1β, IL-6, IL-8, IL-10, and TNF, with **4** showing considerably stronger effects compared to **3**. Notably, **4** additionally induced secretion of IL12p70. Importantly, except for IL-8, the concentrations of the secreted cytokines after stimulation with conjugates were higher than those elicited by the unconjugated mixture of agonists, which indicated that covalent conjugation resulted in amplified immune cell stimulation.

To provide a deeper understanding of the effects induced at the transcriptional level, PBMC mRNA was isolated, amplified, and sequenced after overnight stimulation with **4** as the representative agonist, and the vehicle as the negative control. Differential expression analysis revealed that **4** significantly upregulated the transcription of 962 genes and downregulated the transcription of 877 genes, compared to unstimulated control samples (Figure 3B). Subsequent pathway enrichment analysis of differentially expressed genes using the Kyoto Encyclopedia of Genes and Genomes (KEGG) pathway database revealed the enrichment of several pathways related to the innate immune system (Figure 3C). In line with the results obtained at the protein level (Figure 3A), stimulation with **4** significantly enriched the genes related to cytokine signaling. For instance, **4** strongly upregulated the transcription of interferon γ (IFN-γ) and both IL-12 subunits (IL12A, IL12B), the canonical cytokines of the Th1 immune response.^37,38^ The induction of a Th1-type response was further supported by upregulation of the T-bet transcription factor (Tbx21), which acts as the master regulator of Th1 cell development,^39^ and of IFN-γ–inducible CXCL9, CXCL10, and CXCL11, which function through the CXCR3 receptor expressed on Th1 and natural killer (NK) cells.^40^**4** also induced transcription of IL-17A, IL-17F, IL-22, and IL-26, which together define the secretory phenotype of Th17 cells.^41^ Conversely, the transcription of the prototypical Th2-associated genes (i.e., IL-4, IL-5, IL-13, STAT6, GATA3)^42^ remained unchanged, which indicated that **4** predominantly activated the Th1 and Th17 T-cell subsets. Finally, enrichment of both the “NOD-like receptor signaling” and the “Toll-like receptor signaling” pathways was observed, thus lending further support to the dual PRR activation by **4** (for a detailed list of enriched pathways with their corresponding differentially expressed genes, see Supporting Information, Table S1).

Among the various PBMC subpopulations, NK cells were shown to express both NOD2 and TLR7.^43,44^ In addition to their direct nonspecific cytolytic activity, NK cells also strengthen and direct adaptive immune responses. Specifically, adjuvants that induce NK cell recruitment and activation have been shown to enhance Th1-type immunity, as activated NK cells provide an early source of Th1-polarizing IFN-γ.^45^ Consequently, NK cells have become prominent targets in cancer immunotherapies and vaccine development.^46^

**Figure 4:**
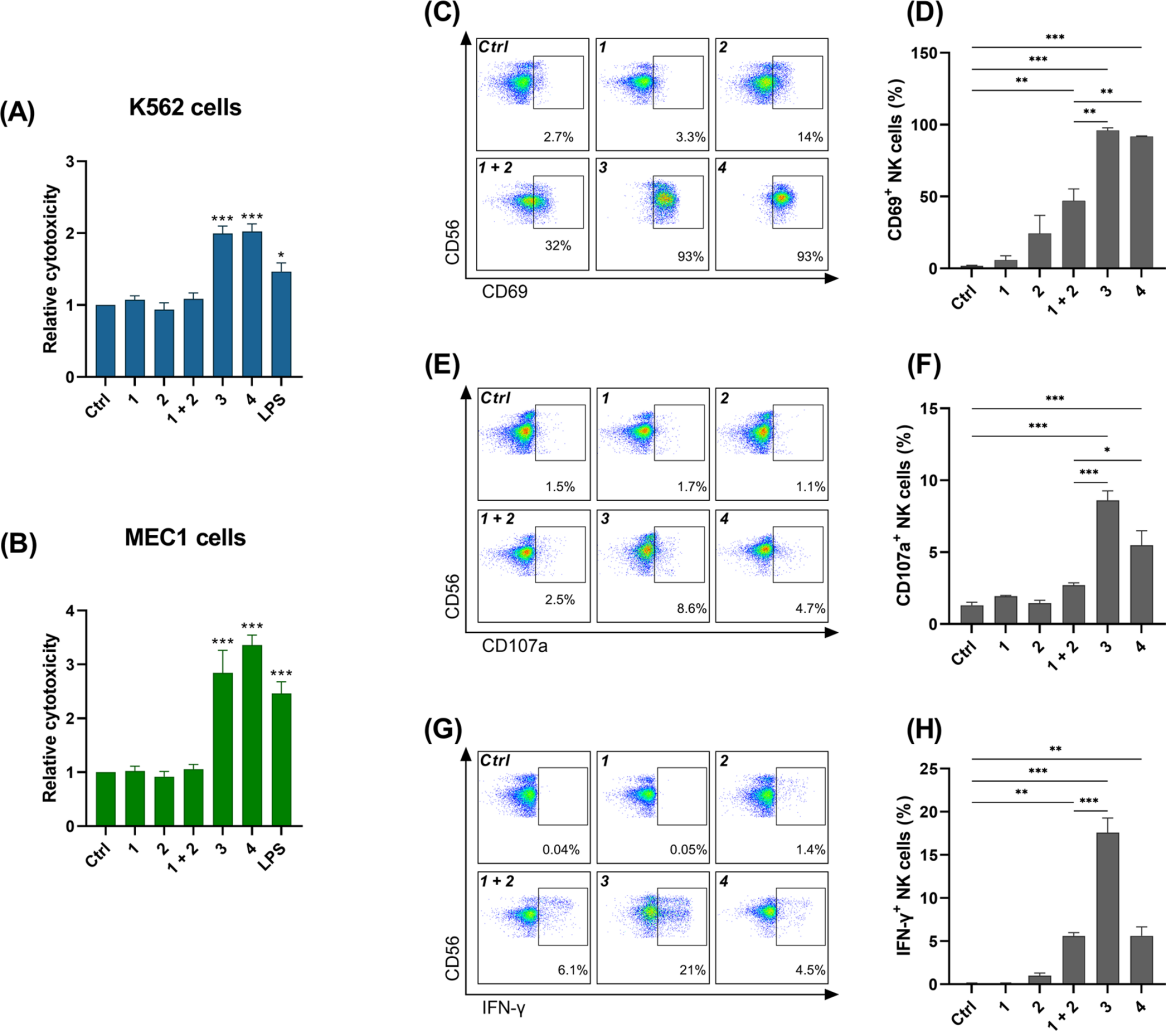
Conjugated NOD2/TLR7 agonists induce cytotoxic activity of PBMCs and activate NK cells. (A, B) Following 18 h stimulation of PBMCs with compounds (1 μM), CFSE-labeled K562 (A) or MEC1 (B) cells were added. Cytotoxicity was determined after 4 h coincubation. Data are shown as relative activities to the vehicle-treated control (0.1% DMSO) and are mean ± SEM of three independent experiments. (C, E, G) Representative dot plots of CD69 (C) and CD107a (E) expression, and IFN-γ production (G) of viable CD3^–^/CD56^+^ NK cells in response to 24 h stimulation of PBMCs with compounds (1 μM). (D, F, H) Pooled data from (C, E, G), expressed as frequencies of CD69^+^, CD107a^+^, and IFN-γ^+^ NK cells. Data are mean ± SEM of three independent experiments. *, *p* < 0.05, **, *p* < 0.01, ***, *p* < 0.001 (one-way ANOVA, *post hoc* Bonferroni’s tests).

The strong induction of IFN-γ transcription by **4** prompted us to investigate whether coengagement of NOD2 and TLR7 results in amplified NK cell responses. We first examined the effects of stimulation with NOD2, TLR7, and NOD2/TLR7 agonists on the nonspecific cytolytic activities of PBMCs against MEC1 and K562 cancer cells. We used the entire PBMC population in lieu of isolated NK cells, to include the contributions of accessory immune cells responsive to NOD2 and TLR7, which interact with NK cells through cytokine secretion.^44,47^ Compounds **3** and **4** significantly enhanced the cytolytic activities of PBMCs against both cancer cell lines (Figure 4A,B). Consistent with the stronger immunostimulatory activity of **4** observed in the PBMC cytokine assay (Figure 3A), **4** also had a significant effect on PBMC cytotoxicity at concentrations as low as 10 nM, while significant activity after stimulation with **3** was observed only at 1 μM (Figure S5). In sharp contrast, single-agonist treatments and co-stimulation with both agonists failed to induce any increases in PBMC cytotoxicity.

To understand how NK cells contributed to the observed PBMC antitumor response, we also examined the expression of the early activation marker CD69, the degranulation marker CD107a, and the production of IFN-γ in the NK cell population (gated as CD3^–^, CD56^+^ cells) in response to stimulation of PBMCs. Both **3** and **4** induced stronger activation and degranulation of NK cells, compared to equimolar concentrations of the individual agonists and their unconjugated mixture (Figure 4C–F). Indeed, individual agonists induced only minor and nonsignificant effects. These effects were stronger after stimulation with both agonists, albeit still much weaker than those induced by either conjugate. Similar synergistic effects were observed after intracellular cytokine staining, with a significant increase of IFN-γ-producing NK cells after stimulation with **3** (Figure 4G,H). Surprisingly, despite the stronger activity of **4** in the functional cytotoxicity assay, compared to **3**, **4** induced a lower increase in IFN-γ-producing NK cells, with comparable activity to the unconjugated mixture of the single agonists.

**Figure 5:**
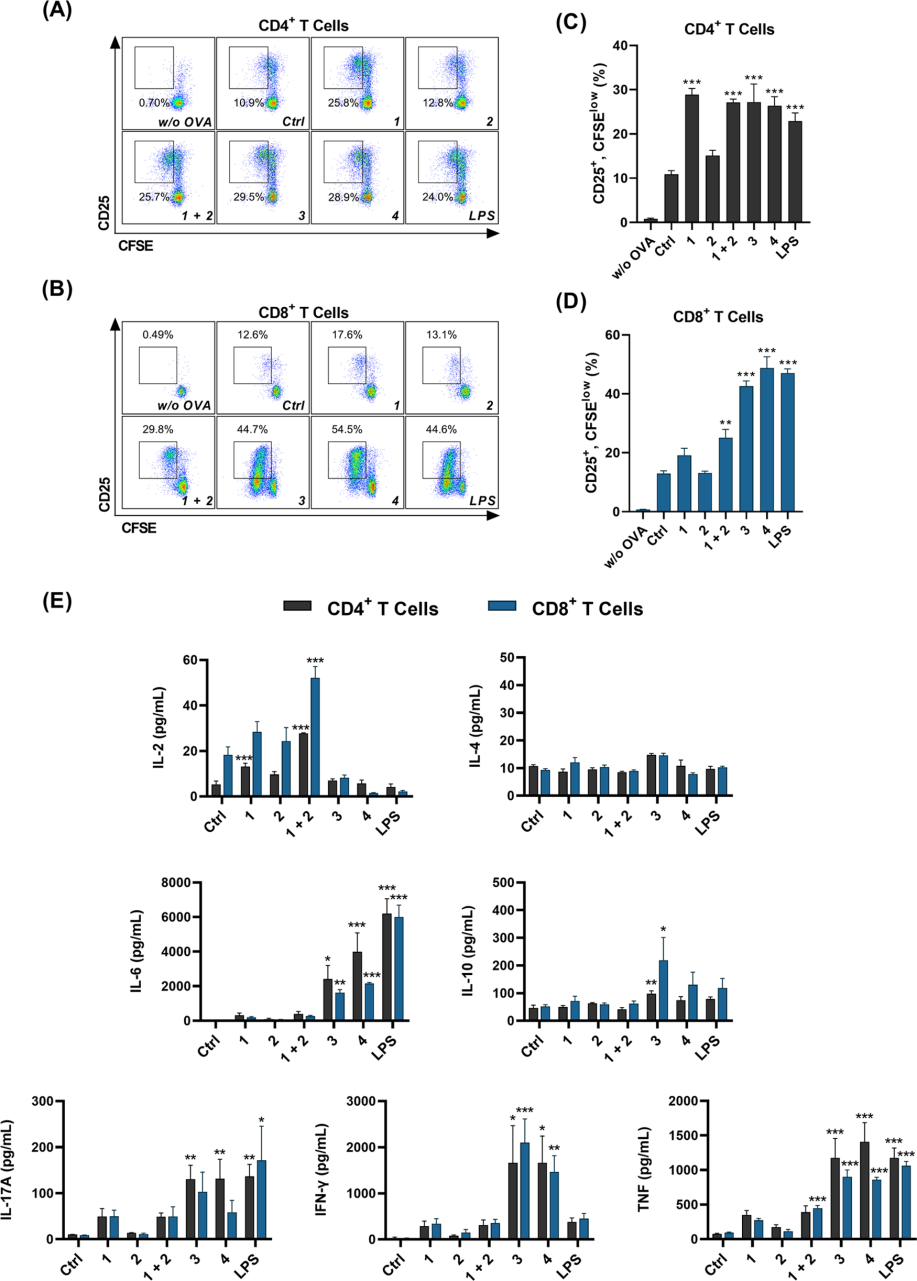
NOD2/TLR7 conjugate treatment of BDMCs promotes their antigen presenting activity and enhances antigen-specific activation and proliferation of CD4^+^ and CD8^+^ T cells. (A–D) BMDCs from C57BL/6 mice were treated for 18 h with compounds (1 μM), LPS (1 μg/mL), or vehicle (0.1% DMSO) in the presence of OVA (50 μg/mL). CFSE-labeled OVA-specific CD4^+^ or CD8^+^ T cells (isolated from OT II or OT I mouse splenocytes, respectively) were added to the treated and washed BMDCs and cocultured for 72 h. (A, B) Representative dot plots show CD25 expression and CFSE dilution in live Thy1.2^+^/CD4^+^ (A) and Thy1.2^+^/CD8^+^ T cells (B). (C, D) Pooled data from (A) and (B), expressed as frequencies of CD25^+^, CFSE^low^ T cells. (E) Cytokine concentrations in BMDC-T-cell coculture supernatants following the 72 h coincubation. Data are mean ± SEM of duplicates of two independent experiments. *, *p* < 0.05, **, *p* < 0.01, ***, *p* < 0.001 *versus* vehicle-treated controls (one-way ANOVA *post hoc* Dunnett’s tests).

After demonstrating the efficacies of these two conjugates in terms of eliciting nonspecific immune responses, we turned our attention toward their enhancement of the development of antigen-specific immunity. An important characteristic of adjuvants is their stimulation of dendritic cells, which in their mature and activated form instruct T and B cells to initiate effective and directed adaptive immune responses.^48^ To investigate how dual stimulation of NOD2 and TLR7 modulates the antigen presentation of dendritic cells, we evaluated the conjugates in a coculture model with murine bone-marrow-derived dendritic cells (BMDCs) and naïve ovalbumin (OVA)-specific CD4^+^ and CD8^+^ T cells, isolated from splenocytes of OT-II and OT-I mice, respectively. BMDCs were treated with immunostimulants and soluble OVA protein, washed, and cocultured for 3 days with carboxyfluorescein succinimidyl ester (CFSE)-labeled T cells. Preactivation of BMDCs with **3** or **4** significantly improved their induction of antigen-specific activation and proliferation of both CD4^+^ and CD8^+^ T cells, measured by upregulation of the early T-cell activation marker CD25 and CFSE dilution (Figure 5A–D). While BMDCs activated by the conjugates had comparable effects on CD4^+^ T-cell activation, the conjugates surpassed the efficacy of the mixture on the activation of CD8^+^ T cells. Subsequent analysis of the cytokine profile in the supernatants of BMDC-T-cell cocultures revealed that stimulation with either conjugate resulted in significantly elevated levels of IL-6, IL-17A, TNF, and IFN-γ (Figure 5E). Consistent with the transcriptomic analysis of **4**-stimulated PBMCs, the enhanced secretion of IFN-γ and IL-17A, along with the undetectable levels of IL-4, are indicative of a Th1/Th17-polarized T-cell response. As opposed to the stronger effects of **4** in PBMCs, comparison between BMDCs activated by **3** and **4** did not reveal any substantial differences in T-cell activation or cytokine secretion. Our results are in agreement with a previous study, which reported that BMDCs stimulated with OVA-loaded nanocapsules adjuvanted with agonists of both NOD2 and TLR7 induced a Th1-biased cytokine profile in T cells, as evidenced by increased secretion of the Th1-associated IFN-γ, while the levels of the Th2-associated IL-5 remained unchanged.^22^

Interestingly, in contrast to the production of the other measured cytokines, the levels of IL-2 followed an inverse trend. While the individual agonists and their unconjugated mixture elicited moderate production of IL-2, stimulation of BMDCs with conjugates or LPS resulted in decreased concentrations of IL-2. During immune responses, IL-2 is consumed in an autocrine or paracrine manner by cells bearing the high-affinity IL-2 receptor (IL-2R).^49^ The expression of CD25, the α subunit of IL-2R, is greatly increased by IL-2, creating a self-reinforcing feedback loop, which consequently also increases the consumption of IL-2. Moreover, although the production of IL-2 by activated T cells is rapid, it is also transient and self-regulating.^50^ The increased expression of CD25 and limited availability of IL-2 observed in our experiments therefore likely reflect the rapid consumption and suppressed production of IL-2 by the highly activated T cells.

**Figure 6:**
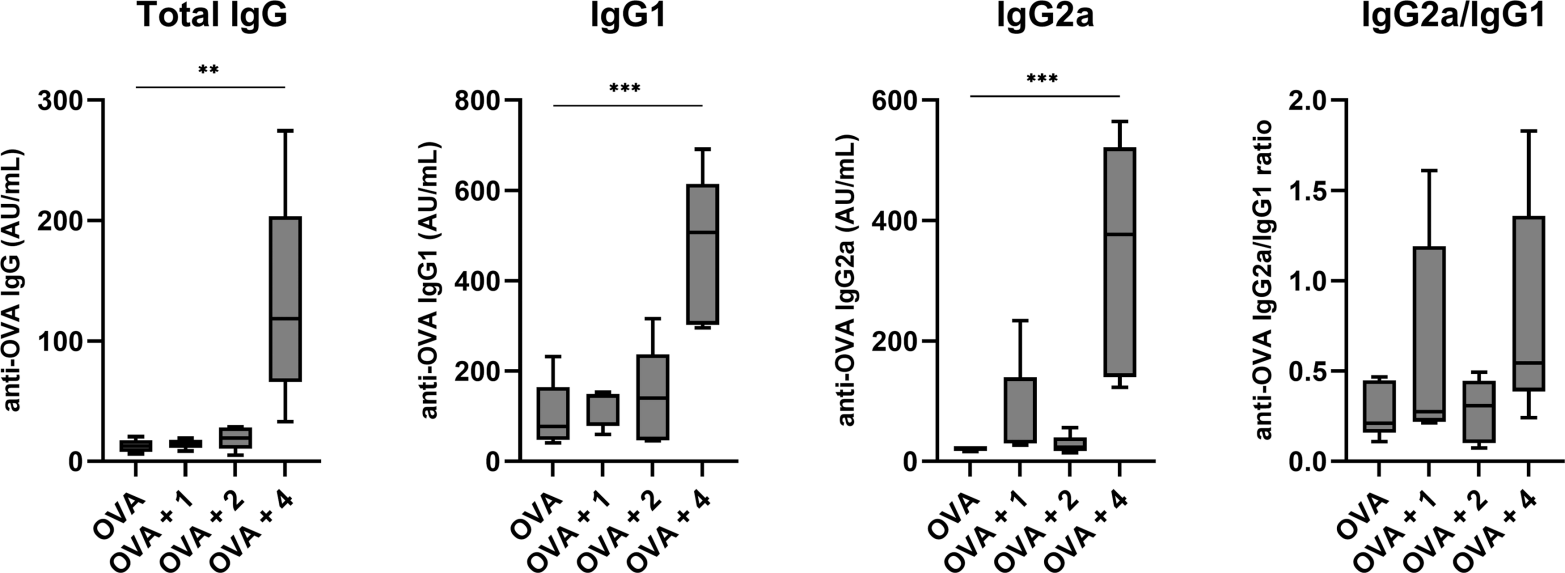
*In vivo* adjuvant activity of NOD2/TLR7 conjugates. NIH/OlaHsd mice (five per group) were immunized s.c. on days 0, 21, and 42 with OVA alone (10 μg) or OVA (10 μg) and the compounds (50 μg). OVA-specific IgG, IgG1, and IgG2a responses were determined 7 days after the last dose. **, *p* < 0.01, ***, *p* < 0.001 *versus* the control group (one-way ANOVA *post hoc* Dunnett’s tests).

Encouraged by the promising *in vitro* results, we next evaluated the adjuvant potential in an *in vivo* murine model of vaccination. Due to the approximately 2-fold higher solubility of **4** (Table S2) and the stronger immunostimulatory activity *in vitro* than seen for **3**, only **4** was used for the *in vivo* evaluation. Following a prime-boost vaccination regimen with OVA as the model antigen and **1**, **2**, and **4** as adjuvants (three doses on days 0, 21, 42), the OVA-specific IgG, IgG1, and IgG2a antibody responses were measured. Compound **4** induced a strong systemic immune response with significantly higher serum OVA-specific IgG titers compared to the mice immunized with OVA alone or immunized with the individual agonists **1** and **2** (Figure 6). Importantly, immunization with **4** increased the titers of both IgG1 and IgG2a antibody isotypes, which serve as indicators of Th2 and Th1 immune responses, respectively,^51^ although the effects observed were stronger on IgG2a. This led to a trend toward increased IgG2a/IgG1 ratios, which indicated that **4** induced a shift toward Th1 immunity, compared to the predominantly Th2-polarized responses generated by either unadjuvanted OVA or the NOD2 agonist **2**. Activation of NOD2 *in vivo* has been previously demonstrated to trigger antigen-specific responses with a Th2-polarized profile, characterized by the production of IL-4 and IL-5 by T cells, and IgG1 antibodies by B cells.^52,53^ However, as was observed in the present study, coengagement of NOD2 with Th1-polarizing TLRs cooperatively enhances the Th1-type responses induced by TLR agonists, although exact mechanisms underlying this phenomenon remain unclear.^52,54^

The data presented here are aligned with the results of a similar NOD2/TLR7 conjugate that was reported during the course of our study.^14^ Here, Gutjahr et al. (2020) reported that dual stimulation of NOD2 and TLR7 by polylactic-acid-encapsulated conjugates induced protective systemic and mucosal immunity *in vivo*. Indeed, in the present study, we observed a shift toward Th1/Th17-type T-cell immunity *in vitro* and toward Th1 humoral immunity *in vivo*. Th1 cells have an instrumental role in the generation of cellular immune responses against intracellular pathogens and tumors. On the other hand, while the Th17 cell subset was first implicated in the development of inflammatory disorders, it is now increasingly recognized as an important contributor to protection against pathogens with a mucosal point of entry.^55^ Given that most currently licensed adjuvants provoke a predominantly Th2-biased response, the induction of Th1- and Th17-biased responses remains a desirable trait in the development of novel vaccine adjuvants. Moreover, the enhanced activities of NK cells and cytotoxic CD8^+^ T cells generated by **3** and **4** further underline the potential value of such compounds in therapeutic vaccines and cancer immunotherapy.

## Conclusions

In conclusion, conjugates with dual NOD2/TLR7 agonist actions, **3** and **4**, were synthesized and biologically evaluated. The potent immunostimulatory activities of both **3** and **4** were demonstrated by enhanced activation of primary human immune cells and mouse dendritic cells, which manifested as potent Th1-biased adjuvant activity of **4***in vivo*. The comparison between **3** and **4** further shows that the chemical nature of the spacer molecule and the attachment site on the NOD2 agonist can significantly affect the physicochemical properties and immunostimulatory capacity of such conjugates and allows for manipulation toward better adjuvant activities. Albeit the lower solubility precluded **3** from progressing to *in vivo* characterization, such physicochemical limitations can easily be avoided by the use of an appropriate delivery system. The benefits of encapsulated over soluble adjuvants have been convincingly demonstrated.^56^ Thus, we plan to further expand the use of our conjugates using the biodegradable polylactic acid nanoparticle platform with the evaluation of these formulations in mucosal vaccines carrying clinically relevant antigens.

This study provides insight into how immune cells respond to multiple input signals and shows that conjugates can generate sophisticated synergistic interactions that individual PRR agonists simply cannot access. Chemical conjugation of multiple PRR agonists thus represents an attractive approach to the development of novel immunopotentiators and may facilitate future vaccine development.

## Experimental Section

### Materials

Chemicals were from Sigma-Aldrich (St. Louis, MO), Tokyo Chemical Industry (Tokyo, Japan), Acros Organics (Geel, Belgium), Enamine (Monmouth Junction, NJ), and Apollo (Stockport, U.K.), and were used without further purification. Lipopolysaccharide (from *E. coli* O55:B5) was from Invivogen, Inc. (San Diego, CA). The TLR7 antagonist enpatoran (**M5049**)^36^ was from MedChemTronica (Sollentuna, Sweden). The NOD2 antagonist **SG84** was synthesized as described previously.^35^ Analytical TLC was performed on Merck 60 F254 silica gel plates (0.25 mm), with visualization using ultraviolet light, ninhydrin, and potassium permanganate. Column chromatography was carried out on silica gel 60 (particle size, 240–400 mesh). ^1^H and ^13^C NMR spectra were recorded at 400 MHz and 100 MHz, respectively, on an Avance III spectrometer (Bruker Corporation, Billerica, MA) in CDCl_3_ or DMSO-*d*_6_ with tetramethylsilane as the internal standard. Mass spectra were obtained using an Exactive Plus orbitrap mass spectrometer (Thermo Fisher Scientific, Waltham, MA) or on Expression CMS mass spectrometer (Advion Inc., Ithaca, NY). Analytical UHPLC analyses were performed on a Dionex UltiMate 3000 Rapid Separation Binary System (Thermo Fisher Scientific, Waltham, MA) equipped with an autosampler, a binary pump system, a photodiode array detector, a thermostated column compartment, and the Chromeleon Chromatography data system. The columns were Waters Acquity UPLC BEH C18 (1.7 μm, 2.1 × 50 mm^2^) or Waters Acquity UPLC CSH C18 (1.7 μm, 2.1 × 50 mm^2^), with a flow rate of 0.3 mL/min. The eluent was a mixture of 0.1% TFA in water (A) and acetonitrile (B), with a gradient of (%B): 0–10 min, 5–95%; 10–12 min, 95%; 12–12.5 min, 95–5%. The columns were thermostated at 40 °C. All of the compounds tested were established to be ≥95% pure.

### General Synthetic Procedures

#### General Procedure A: EDC-Mediated Coupling

To an ice-chilled solution of the requisite amine (1 equiv) and carboxylic acid (1.1–1.2 equiv) in anhydrous dimethylformamide (DMF), *N*,*N*-diisopropylethylamine (DIPEA; 3 equiv), hydroxybenzotriazole (HOBt; 1.1–1.2 equiv), 1-ethyl-3-(3-dimethylaminopropyl)carbodiimide (EDC; 1.1–1.2 equiv), and a catalytic amount of 4-dimethylaminopyridine (DMAP) were added, and the mixture was allowed to warm to room temperature. The stirring was continued overnight, after which the mixture was washed twice with 1 M HCl and saturated NaHCO_3_, and once with brine. The organic layer was dried over anhydrous Na_2_SO_4_ and concentrated in vacuo.

#### General Procedure B: Boc Deprotection

The tert-butyloxycarbonyl (Boc)-protected compound was added to an ice-chilled mixture of trifluoroacetic acid (TFA) and dichloromethane (DCM) (1:5), and the mixture was allowed to warm to room temperature. After 3 h, the solvent was evaporated in vacuo. The residue was washed three times with diethyl ether.

#### Synthesis of TLR7 Agonist **1**(31)

##### 4-((6-Amino-2-chloro-9H-purin-9-yl)methyl)benzonitrile (**5**)

2-Chloroadenine (1.017 g, 6 mmol), potassium carbonate (2.579 g, 18.66 mmol), and 4-(bromomethyl)benzonitrile (1.625 g, 8.29 mmol) were suspended in DMSO (22 mL) and stirred at room temperature for 20 h. The reaction mixture was poured into ethyl acetate (130 mL) and water (90 mL). After concentrating the mixture *in vacuo*, it was cooled in ice and the precipitate formed was filtered, washed with cold water, and dried to give compound **5**. Yield (1.737 g, 89%). ^1^H NMR (400 MHz, DMSO-*d*_6_) δ = 8.28 (s, 1H), 7.92–7.74 (m, 4H), 7.42 (d, *J* = 7.6, 2H), 5.45 (s, 2H).

##### 4-((6-Amino-2-butoxy-9H-purin-9-yl)methyl)benzoic acid (**6**)

Compound **5** (1.737 g, 6.10 mmol) was suspended in dry n-butanol (60 mL). A 20% solution of sodium n-butoxide in n-butanol (33.6 mL, 61.01 mmol) was added, and the resulting mixture was refluxed with stirring for 20 h. The reflux was paused to cool the mixture. Water (20 mL) was added, and the reflux was continued for an additional 20 h. The reaction mixture was extracted three times with 80 mL of water. The combined aqueous layers were acidified to pH 3 with concentrated HCl and cooled overnight. The white precipitate obtained was filtered and dried to give compound **6**. Yield (1.605 g, 77%). ^1^H NMR (400 MHz, DMSO-*d*_6_) δ = 12.96 (s, 1H), 8.07 (s, 1H), 7.91 (d, *J* = 7.1, 2H), 7.38 (d, *J* = 7.1, 2H), 7.26 (s, 2H), 5.35 (s, 2H), 4.24–4.11 (m, 2H), 1.67–1.55 (m, 2H), 1.44–1.33 (m, 2H), 0.90 (t, *J* = 6.7, 3H).

##### 4-((6-Amino-8-bromo-2-butoxy-9H-purin-9-yl)methyl)benzoic acid (**7**)

To a suspension of compound **6** (1.605 g, 4.701 mmol) in acetic acid (60 mL), sodium acetate (1.928 g, 23.51 mmol) and bromine (1.22 mL, 23.74 mmol) were added, and the resulting mixture was stirred at room temperature for 2 h. The reaction was quenched with the addition of aqueous Na_2_S_2_O_3_. The precipitate obtained was filtered and washed with cold water and diethyl ether, to give compound **7** as a yellow powder. Yield (1.976 g, 100%). ^1^H NMR (400 MHz, DMSO-*d*_6_) δ = 7.92 (d, *J* = 8.2, 2H), 7.44 (s, 2H), 7.31 (d, *J* = 8.2, 2H), 5.33 (s, 2H), 4.19 (t, *J* = 6.6, 2H), 1.68–1.58 (m, 2H), 1.43–1.32 (m, 2H), 0.90 (t, *J* = 7.4, 3H).

##### 4-((6-Amino-2-butoxy-8-hydroxy-9H-purin-9-yl)methyl)benzoic acid (**1**)

To a solution of **7** (1.976 g, 4.701 mmol) in methanol (35 mL), 10 M aqueous NaOH (35 mL) was added. The mixture was refluxed with stirring for 24 h. The solution was cooled to room temperature and acidified with 6 M HCl. After concentrating the mixture *in vacuo*, the off-white precipitate obtained was filtered and washed with water and diethyl ether to give compound **1**. Yield (1.523 g, 91%). ^1^H NMR (400 MHz, DMSO-*d*_6_) δ = 10.25 (s, 1H), 7.89 (d, *J* = 8.0, 2H), 7.38 (d, *J* = 8.0, 2H), 6.63 (s, 2H), 4.93 (s, 2H), 4.13 (t, *J* = 6.5, 2H), 1.65–1.54 (m, 2H), 1.40–1.30 (m, 2H), 0.89 (t, *J* = 7.3, 3H).

#### Synthesis of NOD2 Agonists **2** and **18**

##### Diethyl d-glutamate (**8**)

To an ice-chilled stirring suspension of d-glutamic acid (4.414 g, 30 mmol) in absolute ethanol (60 mL), thionyl chloride (4.79 mL, 66 mmol) was added. The resulting mixture was refluxed for 20 h. After concentrating the mixture *in vacuo*, diethyl ether was added to the resulting oily residue to precipitate a solid, which was filtered and washed three times with diethyl ether to give compound **8** as a white crystalline powder. Yield (6.167 g, 86%). ^1^H NMR (400 MHz, DMSO-*d*_6_) δ = 8.71 (s, 2H), 4.19 (q, *J* = 7.1, 2H), 4.07 (q, *J* = 7.1, 2H), 4.01 (t, *J* = 6.7, 1H), 2.62–2.42 (m, 2H), 2.07 (q, *J* = 7.4, 2H), 1.23 (t, *J* = 7.1, 3H), 1.19 (t, *J* = 7.1, 3H).

##### Diethyl (tert-butoxycarbonyl)-l-valyl-d-glutamate (**9**)

Compound **8** (3.596 g, 15 mmol) was coupled to Boc-l-valine (3.585 g, 16.5 mmol) following General Procedure A to give compound **9** as an off-white solid. Yield (5.455 g, 90%). ^1^H NMR (400 MHz, DMSO-*d*_6_) δ = 8.23 (d, *J* = 7.6, 1H), 6.62 (d, *J* = 8.9, 1H), 4.28–4.17 (m, 1H), 4.12–4.00 (m, 4H), 3.84–3.75 (m, 1H), 2.35 (t, *J* = 7.6, 2H), 2.03–1.78 (m, 3H), 1.38 (s, 9H), 1.22–1.13 (m, 6H), 0.84 (t, *J* = 6.6, 6H).

##### Diethyl (tert-butoxycarbonyl)glycyl-l-valyl-d-glutamate (**10**)

Compound **9** (3.699 g, 9.19 mmol) was deprotected using General procedure B and coupled to Boc-glycine (1.771 g, 10.11 mmol) using General Procedure A, to give compound **10** as a yellow oil. Yield (3.644 g, 81%). ^1^H NMR (400 MHz, DMSO-*d*_6_) δ = 8.43 (d, *J* = 7.6, 1H), 7.59 (d, *J* = 9.0, 1H), 7.04 (t, *J* = 6.1, 1H), 4.30–4.19 (m, 2H), 4.14–3.99 (m, 4H), 3.57 (d, *J* = 6.1, 2H), 2.36 (t, *J* = 7.4, 2H), 2.06–1.89 (m, 2H), 1.89–1.74 (m, 1H), 1.38 (s, 9H), 1.20–1.14 (m, 6H), 0.86–0.79 (m, 6H).

##### Diethyl ((E)-3-(4-hydroxy-3-methoxyphenyl)acryloyl)glycyl-l-valyl-d-glutamate (**2**)^30^

Compound **10** (230 mg, 0.5 mmol) was deprotected using General Procedure B and coupled to *trans*-ferulic acid (107 mg, 0.55 mmol) using General Procedure A. Diethyl ether was added to the resulting yellow oil to precipitate a solid, which was filtered and washed twice with diethyl ether to give compound **2** as an orange solid. Yield (128 mg, 48%). ^1^H NMR (400 MHz, DMSO-*d*_6_) δ = 9.45 (s, 1H), 8.39 (d, *J* = 7.5, 1H), 8.20 (t, *J* = 5.7, 1H), 7.89 (d, *J* = 8.9, 1H), 7.33 (d, *J* = 15.7, 1H), 7.14 (d, *J* = 1.8, 1H), 7.00 (dd, *J* = 8.1, 1.8, 1H), 6.79 (t, *J* = 8.1, 1H), 6.56 (d, *J* = 15.7, 1H), 4.30–4.20 (m, 2H), 4.13–3.97 (m, 4H), 3.88 (d, *J* = 6.0, 2H), 3.81 (s, 3H), 2.35 (t, *J* = 7.5, 2H), 2.05–1.90 (m, 2H), 1.89–1.75 (m, 1H), 1.21–1.12 (m, 6H), 0.85 (t, *J* = 6.8, 6H).

##### 5-Benzyl 1-ethyl (tert-butoxycarbonyl)-d-glutamate (**11**)

To an ice-chilled solution of boc-d-glutamic acid 5-benzyl ester (3.374 g, 10 mmol) in DCM (50 mL), DMAP (122 mg, 1 mmol), absolute ethanol (5 mL), HOBt (2.027 g, 15 mmol), and EDC (2.876 g, 15 mmol) were added. The resulting mixture was stirred at room temperature for 18 h. The solvent was evaporated *in vacuo*, and the residue was washed twice with diethyl ether to give compound **11** as a white solid. Yield (2.942 g, 81%). ^1^H NMR (400 MHz, DMSO-*d*_6_) δ = 7.43–7.25 (m, 6H), 5.10 (s, 2H), 4.15–4.02 (m, 2H), 4.02–3.94 (m, 1H), 2.49–2.41 (m, 2H), 2.04–1.92 (m, 1H), 1.89–1.75 (m, 1H), 1.38 (s, 9H), 1.17 (t, *J* = 7.1, 3H).

##### 5-Benzyl 1-ethyl (tert-butoxycarbonyl)-l-valyl-d-glutamate (**12**)

Compound **11** (2.826 g, 7.73 mmol) was deprotected using General Procedure B and coupled to Boc-l-valine (2.016 g, 9.28 mmol) using General Procedure A to give compound **12** as a yellow oil. Yield (3.509 g, 98%). ^1^H NMR (400 MHz, DMSO-*d*_6_) δ = 8.24 (d, *J* = 7.6, 1H), 7.42–7.29 (m, 5H), 6.64 (d, *J* = 9.0, 1H), 5.11–5.06 (m, 2H), 4.30–4.19 (m, 1H), 4.13–4.01 (m, 2H), 3.84–3.74 (m, 1H), 2.44 (t, *J* = 7.6, 2H), 2.10–1.96 (m, 1H), 1.95–1.79 (m, 2H), 1.37 (s, 9H), 1.17 (t, *J* = 7.1, 3H), 0.83 (t, *J* = 6.1, 6H).

##### 5-Benzyl 1-ethyl (tert-butoxycarbonyl)glycyl-l-valyl-d-glutamate (**13**)

Compound **12** (3.306 g, 7.12 mmol) was deprotected using General Procedure B and coupled to Boc-glycine (1.496 g, 8.54 mmol) using General Procedure A, to give compound **13** as a yellow oil. Yield (3.09 g, 83%). ^1^H NMR (400 MHz, DMSO-*d*_6_) δ = 8.45 (d, *J* = 7.6, 1H), 7.60 (d, *J* = 9.0, 1H), 7.42–7.28 (m, 5H), 7.04 (d, *J* = 6.0, 1H), 5.09 (s, 2H), 4.32–4.21 (m, 2H), 4.12–4.02 (m, 2H), 3.57 (d, *J* = 6.0, 2H), 2.44 (t, *J* = 7.5, 2H), 2.08–1.97 (m, 1H), 1.97–1.89 (m, 1H), 1.88–1.79 (m, 1H), 1.37 (s, 9H), 1.16 (t, *J* = 7.1, 3H), 0.86–0.76 (m, 6H).

##### Ethyl (E)-3-(4-hydroxy-3-methoxyphenyl)acrylate (**14**)

To an ice-chilled suspension of *trans*-ferulic acid (2.913 g, 15 mmol) in absolute ethanol (30 mL), thionyl chloride (1.31 mL, 18 mmol) was added. The resulting mixture was refluxed for 20 h. The solution was concentrated *in vacuo*, dissolved in ethyl acetate (40 mL), and washed with 1 M HCl (25 mL), saturated NaHCO_3_ (20 mL), and brine (20 mL). The organic layer was dried over anhydrous Na_2_SO_4_ and concentrated *in vacuo* to give compound **14** as an orange oil. Yield (3.218 g, 97%). ^1^H NMR (400 MHz, DMSO-*d*_6_) δ = 9.61 (s, 1H), 7.54 (d, *J* = 15.9, 1H), 7.33 (d, *J* = 2.0, 1H), 7.12 (dd, *J* = 8.2, 2.0, 1H), 6.79 (d, *J* = 8.2, 1H), 6.48 (d, *J* = 15.9, 1H), 4.16 (q, *J* = 7.1, 2H), 3.81 (s, 3H), 1.25 (t, *J* = 7.1, 3H).

##### Ethyl (E)-3-(3-methoxy-4-((tetrahydro-2H-pyran-2-yl)oxy)phenyl)acrylate (**15**)

To a solution of compound **14** (3.195 g, 14.38 mmol) in 3,4-dihydropyran (30 mL), pyridinium p-toluenesulfonate (0.291 g, 1.16 mmol) was added. The resulting mixture was refluxed for 2 h. The solution was concentrated *in vacuo*, dissolved in ethyl acetate (50 mL), and washed with 1 M NaOH (5× 10 mL), water (20 mL), and brine (20 mL). The solvent was evaporated *in vacuo* to give compound **15** as an orange oil. Yield (4.327 g, 98%). ^1^H NMR (400 MHz, DMSO-*d*_6_) δ = 7.58 (d, *J* = 16.0, 1H), 7.39 (d, *J* = 2.1, 1H), 7.22 (dd, *J* = 8.4, 2.1, 1H), 7.09 (d, *J* = 8.4, 1H), 6.58 (d, *J* = 16.0, 1H), 5.49 (t, *J* = 3.3, 1H), 4.18 (q, *J* = 7.1, 2H), 3.83 (s, 3H), 3.81–3.72 (m, 1H), 3.60–3.50 (m, 1H), 1.96–1.70 (m, 3H), 1.64–1.41 (m, 3H), 1.26 (t, *J* = 7.1, 3H).

##### (E)-3-(3-Methoxy-4-((tetrahydro-2H-pyran-2-yl)oxy)phenyl)acrylic acid (**16**)^57^

To a solution of compound **15** (4.303 g, 14.04 mmol) in methanol (30 mL), 1 M aqueous NaOH (20 mL) was added. The resulting mixture was stirred at room temperature for 20 h. The mixture was concentrated *in vacuo*, and the residue was dissolved in water (20 mL) and washed with ethyl acetate (2 × 30 mL). The aqueous layer was acidified with 1 M HCl and extracted with ethyl acetate (2 × 50 mL). The combined organic phases were washed with water (50 mL) and brine (50 mL), dried over anhydrous Na_2_SO_4_, and concentrated *in vacuo*, to give compound **16** as a white solid. Yield (2.508 g, 64%). ^1^H NMR (400 MHz, DMSO-*d*_6_) δ = 12.25 (s, 1H), 7.53 (d, *J* = 16.0, 1H), 7.35 (d, *J* = 2.5, 1H), 7.18 (dd, *J* = 8.4, 2.5, 1H), 7.10 (d, *J* = 8.4, 1H), 6.46 (d, *J* = 16.0, 1H), 5.48 (t, *J* = 3.4, 1H), 3.83 (s, 3H), 3.81–3.74 (m, 1H), 3.59–3.49 (m, 1H), 1.93–1.73 (m, 3H), 1.67–1.48 (m, 3H).

##### 5-Benzyl 1-ethyl ((E)-3-(3-methoxy-4-((tetrahydro-2H-pyran-2-yl)oxy)phenyl)acryloyl)glycyl-l-valyl-d-glutamate (**17**)

Compound **13** (862 mg, 1.65 mmol) was deprotected using General Procedure B and coupled to compound **16** (506 mg, 1.82 mmol) using General Procedure A (a saturated NH_4_Cl solution was used instead of 1 M HCl in the washing step to preserve the tetrahydropyranyl protecting group). The crude product was purified using column chromatography (MeOH/DCM 1:25) to give compound **17** as an off-white solid. Yield (844 mg, 75%). *R*_f_ = 0.19 (MeOH/DCM 1:25). ^1^H NMR (400 MHz, DMSO-*d*_6_) δ = 8.40 (d, *J* = 7.6, 1H), 8.25 (d, *J* = 5.8, 1H), 7.92 (d, *J* = 9.0, 1H), 7.42–7.28 (m, 6H), 7.21 (s, 1H), 7.09 (s, 2H), 6.65 (d, *J* = 15.8, 1H), 5.46 (t, *J* = 3.3, 1H), 5.06 (s, 2H), 4.33–4.20 (m, 2H), 4.12–4.01 (m, 2H), 3.90 (d, *J* = 5.8, 2H), 3.82 (s, 3H), 3.80–3.74 (m, 1H), 3.58–3.48 (m, 1H), 2.43 (t, *J* = 7.5, 2H), 2.08–1.92 (m, 2H), 1.92–1.71 (m, 4H), 1.67–1.48 (m, 3H), 1.17 (t, *J* = 7.2, 3H), 0.84 (t, *J* = 6.3, 6H).

##### (4R)-5-Ethoxy-4-((2S)-2-(2-((E)-3-(3-methoxy-4-((tetrahydro-2H-pyran-2-yl)oxy)phenyl)acrylamido)acetamido)-3-methylbutanamido)-5-oxopentanoic acid (**18**)

To a solution of palladium (II) acetate (12 mg, 0.055 mmol) in dry DCM (7 mL), Et_3_N (297 μL, 2.13 mmol) and triethylsilane (350 μL, 2.19 mmol) were added. The resulting black solution was stirred at room temperature for 15 min, after which a solution of compound **17** (746 mg, 1.09 mmol) in dry DCM (13 mL) was added. The resulting mixture was stirred under argon atmosphere at room temperature for 18 h, after which it was diluted with DCM (30 mL) and extracted with water (3 × 30 mL). The combined aqueous phases were acidified with 1 M HCl and extracted with DCM (2 × 50 mL). The combined organic phases were washed with water (2 × 50 mL) and brine (50 mL), dried over anhydrous Na_2_SO_4_, and concentrated *in vacuo* to give compound **18** as a white solid. Yield (355 mg, 55%). ^1^H NMR (400 MHz, DMSO-*d*_6_) δ = 12.17 (s, 1H), 8.56–8.44 (m, 1H), 8.42–8.32 (m, 1H), 7.88 (d, *J* = 9.0, 1H), 7.37 (d, *J* = 15.6, 1H), 7.22 (s, 1H), 7.10 (s, 2H), 6.68 (d, *J* = 15.6, 1H), 5.45 (t, *J* = 3.3, 1H), 4.30–4.19 (m, 2H), 4.12–4.03 (m, 2H), 3.90 (d, *J* = 5.7, 2H), 3.82 (s, 3H), 3.81–3.74 (m, 1H), 3.58–3.49 (m, 1H), 2.27 (t, *J* = 7.3, 2H), 2.07–1.70 (m, 6H), 1.66–1.50 (m, 3H), 1.18 (t, *J* = 7.1, 3H), 0.85 (t, *J* = 6.4, 6H).

#### Synthesis of Mono-Protected Linkers

##### 6-Ethoxy-6-oxohexan-1-aminium chloride (**19**)

To a solution of 6-aminohexanoic acid (656 mg, 5 mmol) in absolute ethanol (5 mL), thionyl chloride (0.55 mL, 7.5 mmol) was added. The resulting mixture was refluxed for 3 h. The reaction mixture was concentrated *in vacuo* and coevaporated with diethyl ether to give compound **19** as a white solid. Yield (976 mg, 100%). ^1^H NMR (400 MHz, DMSO-*d*_6_) δ = 8.00 (s, 3H), 4.05 (q, *J* = 7.1, 2H), 2.74 (t, *J* = 7.8, 2H), 2.28 (t, *J* = 7.3, 2H), 1.61–1.47 (m, 4H), 1.37–1.26 (m, 2H), 1.18 (t, *J* = 7.1, 3H).

##### tert-Butyl(2-(2-aminoethoxy)ethyl)carbamate (**20**)

Compound **20** was synthesized from 2-(2-aminoethoxy)ethan-1-ol as described previously.^58^

#### Synthesis of NOD2/TLR7 Conjugates **3** and **4**

##### Ethyl 6-(4-((6-amino-2-butoxy-8-hydroxy-9H-purin-9-yl)methyl)benzamido)hexanoate (**21**)

Compound **1** (200 mg, 0.56 mmol) was dissolved in DMSO (8 mL). Compound **19** (329 mg, 1.68 mmol) and DIPEA (780 μL, 4.48 mmol) were dissolved in DCM (2 mL) and added to the stirring solution of compound **1** in DMSO. After cooling the reaction mixture in ice, COMU (600 mg, 1.40 mmol) was added, and the mixture was stirred at room temperature for 20 h. Ethyl acetate (60 mL) and 1 M NaHCO_3_ (40 mL) were added. After concentrating the mixture *in vacuo*, it was cooled in ice for 1 h. The precipitate obtained was filtered and washed with water and diethyl ether to give compound **21** as a tan solid. Yield (158 mg, 57%). ^1^H NMR (400 MHz, DMSO-*d*_6_) δ = 9.99 (s, 1H), 8.40 (t, *J* = 5.7, 1H), 7.77 (d, *J* = 8.4, 2H), 7.35 (d, *J* = 8.4, 2H), 6.48 (s, 2H), 4.90 (s, 2H), 4.19–4.10 (m, 2H), 4.02 (q, *J* = 7.1, 2H), 3.25–3.17 (m, 2H), 2.27 (t, *J* = 7.4, 2H), 1.66–1.56 (m, 2H), 1.56–1.45 (m, 4H), 1.36 (q, *J* = 7.4, 2H), 1.32–1.25 (m, 2H), 1.15 (t, *J* = 7.1, 3H), 0.90 (t, *J* = 7.4, 3H).

##### 6-(4-((6-Amino-2-butoxy-8-hydroxy-9H-purin-9-yl)methyl)benzamido)hexanoic acid (**22**)

To a stirring solution of compound **21** (150 mg, 0.30 mmol) in methanol (10 mL), 1 M aqueous NaOH (3 mL) was added. The mixture was stirred at room temperature for 20 h. Water (12 mL) was added, and the mixture was acidified with 1 M HCl. Methanol was evaporated *in vacuo*, and the resulting suspension was cooled in ice for 1 h. The precipitate obtained was filtered and washed with water and diethyl ether to give compound **22** as an off-white solid. Yield (76 mg, 54%). ^1^H NMR (400 MHz, DMSO-*d*_6_) δ = 11.98 (s, 1H), 9.97 (s, 1H), 8.39 (t, *J* = 5.6, 1H), 7.77 (d, *J* = 8.2, 2H), 7.34 (d, *J* = 8.2, 2H), 6.46 (s, 2H), 4.90 (s, 2H), 4.13 (t, *J* = 6.7, 2H), 3.22 (q, *J* = 6.7, 2H), 2.19 (t, *J* = 7.3, 2H), 1.61 (p, *J* = 6.7, 2H), 1.55–1.44 (m, 4H), 1.42–1.24 (m, 4H), 0.90 (t, *J* = 7.4, 3H).

##### Diethyl ((E)-3-(4-((6-(4-((6-amino-2-butoxy-8-hydroxy-9H-purin-9-yl)methyl)benzamido)hexanoyl)-oxy)-3-methoxyphenyl)acryloyl)glycyl-l-valyl-d-glutamate (**3**)

To an ice-chilled stirring solution of compound **22** (55 mg, 0.12 mmol) in DMF (2 mL), DIPEA (61 μL, 0.35 mmol), compound **2** (63 mg, 0.12 mmol), and COMU (55 mg, 0.13 mmol) were added. The resulting mixture was stirred at room temperature for 4 h, after which it was diluted with ethyl acetate (30 mL) and washed with 1 M HCl (2 × 15 mL), saturated NaHCO_3_ (2 × 15 mL), and brine (15 mL). The organic layer was dried over anhydrous Na_2_SO_4_ and concentrated *in vacuo*. The crude compound was purified using column chromatography (MeOH/DCM 1:15), to give compound **3** as an orange solid. Yield (14 mg, 12%). *R*_f_ = 0.16 (MeOH/DCM 1:15). ^1^H NMR (400 MHz, DMSO-*d*_6_) δ = 10.02 (s, 1H), 8.47–8.37 (m, 2H), 8.33 (t, *J* = 5.7, 1H), 7.95 (d, *J* = 9.0, 1H), 7.78 (d, *J* = 8.0, 2H), 7.42 (d, *J* = 15.9, 1H), 7.37–7.30 (m, 3H), 7.19–7.06 (m, 2H), 6.78 (d, *J* = 15.9, 1H), 6.49 (s, 2H), 4.91 (s, 2H), 4.31–4.21 (m, 2H), 4.19–3.97 (m, 6H), 3.92 (d, *J* = 5.7, 2H), 3.79 (s, 3H), 3.30–3.21 (m, 2H), 2.57 (t, *J* = 7.3, 2H), 2.36 (t, *J* = 7.5, 2H), 2.05–1.94 (m, 2H), 1.91–1.77 (m, 1H), 1.71–1.47 (m, 6H), 1.45–1.31 (m, 4H), 1.20–1.11 (m, 6H), 0.93–0.82 (m, 9H). ^13^C NMR (100 MHz, DMSO-*d*_6_) δ = 172.54, 172.01, 171.49, 169.31, 166.25, 165.73, 160.57, 152.69, 151.52, 149.60, 148.28, 140.71, 140.53, 138.90, 134.31, 134.29, 127.83, 127.66, 123.69, 122.57, 120.59, 112.05, 98.73, 66.31, 61.05, 60.39, 57.83, 56.23, 51.54, 42.72, 42.58, 40.79, 33.60, 31.26, 31.04, 30.19, 29.25, 26.39, 26.24, 24.71, 19.58, 19.20, 18.23, 14.52, 14.47, 14.16. HRMS *m*/*z* calculated for C_49_H_66_O_13_N_9_: 988.4775 (M + H)^+^, found 988.4779.

##### tert-Butyl (2-(2-(4-((6-amino-2-butoxy-8-hydroxy-9H-purin-9-yl)methyl)benzamido)ethoxy)ethyl)carbamate (**23**)

Compound **1** (550 mg, 1.54 mmol) was dissolved in DMSO (8 mL). Compound **20** (944 mg, 4.62 mmol) and DIPEA (2.15 mL, 12.3 mmol) were dissolved in DCM (2 mL) and added to the stirring solution of compound **1** in DMSO. After cooling the reaction mixture in ice, COMU (1.649 g, 3.85 mmol) was added, and the mixture was stirred at room temperature for 20 h. Ethyl acetate (60 mL) and 1 M NaHCO_3_ (40 mL) were added. After concentrating the mixture *in vacuo*, it was cooled in ice for 1 h. The precipitate obtained was filtered and washed with water and diethyl ether to give compound **23** as an off-white solid. Yield (658 mg, 79%). ^1^H NMR (400 MHz, DMSO-*d*_6_) δ = 9.98 (s, 1H), 8.44 (t, *J* = 6.4, 1H), 7.78 (d, *J* = 8.2, 2H), 7.34 (d, *J* = 8.2, 2H), 6.76 (t, *J* = 6.8, 1H), 6.47 (s, 2H), 4.90 (s, 2H), 4.12 (t, *J* = 7.0, 2H), 3.53–3.45 (m, 2H), 3.43–3.36 (m, 4H), 3.14 – 2.97 (m, 2H), 1.69–1.52 (m, 2H), 1.42–1.27 (m, 11H), 0.89 (t, *J* = 7.4, 3H).

##### 4-((6-Amino-2-butoxy-8-hydroxy-9H-purin-9-yl)methyl)-N-(2-(2-aminoethoxy)ethyl)benzamide (**24**)

Compound **23** (650 mg, 1.20 mmol) was deprotected using General procedure B to give compound **24** as a brown solid. Yield (663 mg, 99%). ^1^H NMR (400 MHz, DMSO-*d*_6_) δ = 10.08 (s, 1H), 8.43 (t, *J* = 5.8, 1H), 7.84–7.66 (m, 5H), 7.36 (d, *J* = 8.2, 2H), 6.53 (s, 2H), 4.91 (s, 2H), 4.13 (t, *J* = 7.1, 2H), 3.64–3.52 (m, 4H), 3.50–3.40 (m, 2H), 3.04–2.93 (m, 2H), 1.68–1.55 (m, 2H), 1.44–1.29 (m, 2H), 0.90 (t, *J* = 7.5, 3H).

##### Ethyl N5-(2-(2-(4-((6-amino-2-butoxy-8-hydroxy-9H-purin-9-yl)methyl)benzamido)ethoxy)ethyl)-N2-((E)-3-(4-hydroxy-3-methoxyphenyl)acryloyl)glycyl-l-valyl-d-glutaminate (**4**)

To an ice-chilled stirring solution of compound **18** (71 mg, 0.12 mmol) in DMF (3 mL), DIPEA (84 μL, 0.48 mmol), compound **24** (115 mg, 0.21 mmol), and 1-[bis(dimethylamino)methylene]-1H-1,2,3-triazolo[4,5-*b*]pyridinium 3-oxide hexafluorophosphate (HATU; 64 mg, 0.17 mmol) were added. The resulting mixture was stirred at room temperature for 20 h. Subsequently, 1 M HCl (20 mL) was added, and the mixture was stirred at room temperature for 15 min (to remove the tetrahydropyranyl protecting group), after which it was extracted with a mixture of DCM and isopropanol (3/1, 3× 20 mL). The combined organic phases were washed with saturated NaHCO_3_ (2× 50 mL) and brine (50 mL), dried over anhydrous Na_2_SO_4_, and concentrated *in vacuo*. The crude product was purified using column chromatography (MeOH/DCM 1:9), to give compound **4** as a white solid. Yield (48 mg, 39%). *R*_f_ = 0.14 (MeOH/DCM 1:9). ^1^H NMR (400 MHz, DMSO-d_6_) δ = 9.98 (s, 1H), 9.44 (s, 1H), 8.48 (t, *J* = 5.6, 1H), 8.28 (d, *J* = 8.2, 1H), 8.16 (t, *J* = 5.9, 1H), 7.97 (d, *J* = 8.3, 1H), 7.91 (t, *J* = 5.7, 1H), 7.78 (d, *J* = 8.3, 2H), 7.38–7.30 (m, 3H), 7.14 (d, *J* = 2.2, 1H), 7.00 (dd, *J* = 8.2, 2.2, 1H), 6.79 (d, *J* = 8.2, 1H), 6.55 (d, *J* = 15.8, 1H), 6.46 (d, *J* = 6.4, 2H), 4.90 (s, 2H), 4.29–4.09 (m, 4H), 4.01 (q, *J* = 7.0, 2H), 3.88 (d, *J* = 6.0, 2H), 3.80 (s, 3H), 3.52–3.44 (m, 4H), 3.41–3.36 (m, 2H), 3.27–3.10 (m, 2H), 2.32–2.23 (m, 2H), 2.01–1.87 (m, 2H), 1.81–1.67 (m, 1H), 1.66–1.55 (m, 2H), 1.43–1.30 (m, 2H), 1.14 (t, *J* = 7.2, 3H), 0.89 (t, *J* = 7.4, 3H), 0.84 (d, *J* = 6.8, 6H). ^13^C NMR (100 MHz, DMSO-*d*_6_) δ = 172.24, 171.68, 171.48, 169.48, 166.49, 166.27, 160.57, 152.67, 149.58, 148.81, 148.27, 148.26, 140.69, 139.94, 133.97, 127.88, 127.65, 126.78, 122.07, 118.95, 116.09, 111.32, 98.72, 69.34, 69.16, 66.30, 60.94, 57.81, 55.96, 52.18, 42.71, 42.58, 38.98, 31.77, 31.26, 31.03, 27.12, 19.65, 19.20, 18.20, 14.49, 14.17. HRMS *m*/*z* calculated for C_45_H_61_O_12_N_10_: 933.4465 (M + H)^+^, found 933.4458.

### Solubility Measurement

The kinetic solubilities of conjugates **3** and **4** were estimated using the method described by Hoelke et al.^59^ Briefly, in duplicate, a 10 mM solution of compound in DMSO was diluted with pH 7.4 phosphate-buffered saline (PBS) to give a final DMSO concentration of 2%. The resulting suspension was shaken for 2 h at room temperature, filtered through a syringe filter (0.45 μM), diluted 2-fold with acetonitrile, and analyzed with UHPLC. The solubility was quantified with a five-point calibration curve from 500 to 0.4 μM generated by dilution of the original DMSO solution into a 1:1 mixture of acetonitrile and PBS. The content of DMSO in all solutions was fixed to 2% by adding the respective amounts of DMSO. The column used was Waters Acquity UPLC BEH C18 (1.7 μm, 2.1 × 50 mm^2^). The eluent was a mixture of 0.1% TFA in water (A) and acetonitrile (B), with a gradient of (%B): 0–6 min, 5–95%; 6–8 min, 95%; 8–8.5 min, 95–5%. The column was thermostated at 40 °C.

### Mice

#### Experiments with Bone-Marrow-Derived Dendritic Cells and T Cells

C57BL/6, OT I (C57BL/6-Tg(TcraTcrb)1100Mjb/J), and OT II (C57BL/6-Tg(TcraTcrb)425Cbn/Crl) mice were purchased from Jackson Laboratory (Bar Harbor, ME) and bred at the University of Leiden (The Netherlands). The mice were kept under standard laboratory conditions, with food and water provided *ad libitum*. The mice were euthanized while sedated, by cervical dislocation. All animal work was performed according to the guidelines of the European Parliament Directive 2010/63EU, and the experimental work was approved by the Animal Ethics Committee of Leiden University. For culture conditions of BMDCs, see below.

#### In Vivo Experiments

NIH/OlaHsd inbred mice were raised at the Institute of Immunology, Croatia. All mice used were females, from 2.0 to 2.5 months old. During the experimental period, the mice were housed in the Animal Facility of the Institute of Immunology, with food and water provided *ad libitum*. All animal work was performed according to the Croatian Law on Animal Welfare (2017), which complies strictly with the EC Directive (2010/63/EU).

### Cell Culture

#### HEK-Blue NOD1, NOD2, and TLR7 Cells

HEK-Blue NOD1 (Cat. code: hkb-hnod1), NOD2 (Cat. code: hkb-hnod2), and TLR7 (Cat. code: hkb-htlr7) cell lines (Invivogen, San Diego, CA) are derived from HEK293 cells by co-transfection of the hNOD1, hNOD2, or hTLR7 genes, respectively, and a nuclear factor-κB (NF-κB)-inducible secreted embryonic alkaline phosphatase (SEAP) reporter gene. Following activation of the respective receptors, the resulting NF-κB induces the production of SEAP, the levels of which can be quantified colorimetrically. HEK-Blue cells were cultured according to the manufacturer instructions, in Dulbecco’s modified Eagle’s medium (Sigma-Aldrich, St. Louis, MO) supplemented with 10% heat-inactivated fetal bovine serum (Gibco), 2 mM l-glutamine (Sigma-Aldrich), 100 U/mL penicillin (Sigma-Aldrich), 100 μg/mL streptomycin (Sigma-Aldrich), and 100 μg/mL Normocin (Invivogen) for two passages. All of the subsequent passages were cultured in medium additionally supplemented with 100 μg/mL Zeocin (Invivogen) and 30 μg/mL Blasticidin (Invivogen) for HEK-Blue NOD1 and NOD2 cells, or with 100 μg/mL Zeocin (Invivogen) and 10 μg/mL Blasticidin (Invivogen) for HEK-Blue TLR7 cells. The cells were incubated in a humidified atmosphere at 37 °C and 5% CO_2_.

#### RAW-Blue Cells

RAW-Blue cells (Cat. code: raw-sp; Invivogen, San Diego, CA) were cultured according to the manufacturer’s instructions, in Dulbecco’s modified Eagle’s medium (Sigma-Aldrich, St. Louis, MO) supplemented with 10% heat-inactivated fetal bovine serum (Gibco), 2 mM l-glutamine (Sigma-Aldrich), 100 U/mL penicillin (Sigma-Aldrich), 100 μg/mL streptomycin (Sigma-Aldrich), and 100 μg/mL Normocin (Invivogen). After the first two passages, 200 μg/mL Zeocin (Invivogen) was added to the medium every other passage, to maintain the selection pressure. The cells were incubated in a humidified atmosphere at 37 °C and 5% CO_2_.

#### Peripheral Blood Mononuclear Cells

Human PBMCs from healthy and consenting donors were isolated from heparinized blood by density gradient centrifugation using Ficoll-Paque (Pharmacia, Sweden). The isolated cells were washed twice with PBS, resuspended in RPMI 1640 medium (Sigma-Aldrich, St. Louis, MO) supplemented with 10% heat-inactivated fetal bovine serum (Gibco), 2 mM l-glutamine (Sigma-Aldrich), 100 U/mL penicillin (Sigma-Aldrich), and 100 μg/mL streptomycin (Sigma-Aldrich), and used in the assays.

#### Cancer Cell Lines

K562 cells are a chronic myelogenous leukemia cell line (Cat. code: CCL-243; ATCC, Manassas, VA)^60^ and MEC1 cells are a B-chronic lymphocytic leukemia cell line (Cat. code: ACC 497; DSMZ GmbH, Braunschweig, Germany).^61^ K562 cells were cultured in RPMI 1640 medium (Sigma-Aldrich, St. Louis, MO) supplemented with 10% heat-inactivated fetal bovine serum (Gibco), 2 mM l-glutamine (Sigma-Aldrich), 100 U/mL penicillin (Sigma-Aldrich), and 100 μg/mL streptomycin (Sigma-Aldrich). MEC1 cells were cultured in Iscove’s modified Dulbecco’s medium supplemented with 10% heat-inactivated fetal bovine serum (Gibco), 2 mM l-glutamine (Sigma-Aldrich), 100 U/mL penicillin (Sigma-Aldrich), and 100 μg/mL streptomycin (Sigma-Aldrich). The cells were incubated in a humidified atmosphere at 37 °C and 5% CO_2_.

#### Bone-Marrow-Derived Dendritic Cells

Bone-marrow cells were isolated from the tibia of C57BL/6 mice and cultured in Dulbecco’s modified Eagle’s medium (Lonza, Basel, Switzerland) supplemented with 10% heat-inactivated fetal bovine serum (Lonza), 2 mM l-glutamine (Lonza), 100 U/mL penicillin (Lonza), 100 μg/mL streptomycin (Lonza), and 20 ng/mL granulocyte-macrophage colony-stimulating factor (ImmunoTools, Friesoythe, Germany) for 7 days at 37 °C and 5% CO_2_. The purity of the BMDCs was evaluated with a PE-labeled anti-mouse CD11c antibody (Biolegend, San Diego, CA) by flow cytometry, with > 90% shown to be CD11c-positive.

### Cytotoxicity

The tested compounds were dissolved in DMSO and further diluted in culture medium to the desired final concentrations such that the final DMSO concentration never exceeded 0.1%. HEK-Blue NOD2 cells (40,000 cells/well), RAW-Blue cells (100,000 cells/well), or PBMCs (200.000 cells/well) were seeded in duplicate in 96-well plates in 100 μL of culture medium and treated with 10 μM of each compound or with the corresponding vehicle (0.1% DMSO). After 18 h of incubation (37 °C, 5% CO_2_), the metabolic activity was assessed using CellTiter 96 Aqueous One Solution cell proliferation assays (Promega, Madison, WI), according to the manufacturer’s instructions.

### Measurement of NF-κB Transcriptional Activity in HEK-Blue Cells

HEK-Blue NOD1, NOD2. or TLR7 cells were seeded (25,000 cells/well) in duplicate in 96-well plates in 100 μL of HEK-Blue detection medium (Invivogen, San Diego, CA) and treated in duplicates with the compounds (10 μM for fixed concentration assays; eight different concentrations ranging from 1 nM to 10 μM for determination of EC_50_) or with the corresponding vehicle (0.1% DMSO). After 18 h of incubation (37 °C, 5% CO_2_), SEAP activity was determined spectrophotometrically as absorbance at 630 nm (BioTek Synergy microplate reader; Winooski, VT). EC_50_ values were calculated using the Prism software (version 9; GraphPad Software, CA).

### Measurement of NF-κB Transcriptional Activity in RAW-Blue Cells

RAW-Blue cells were seeded (100,000 cells/well) in duplicate in 96-well plates in 200 μL of growth medium (without Normocin and Zeocin) and pretreated with a NOD2 antagonist **SG84** (5 μM),^35^ TLR7 antagonist **M5049** (1 μM),^36^ or both for 1 h, before the addition of compounds (1 μM) or the corresponding vehicle (0.1% DMSO). After 18 h of incubation (37 °C, 5% CO_2_), 10 μL of the induced supernatant was added to 90 μL of QUANTI-Blue solution (Invivogen). After 2 h of incubation (37 °C), SEAP activity was determined spectrophotometrically as absorbance at 630 nm (BioTek Synergy microplate reader; Winooski, VT). Statistical significance was determined with two-way ANOVA with subsequent Dunnett’s multiple comparisons test.

### Cytokine Release from Peripheral Blood Mononuclear Cells

Peripheral blood mononuclear cells were seeded (1 × 10^6^ cells/mL) in 24-well plates in 500 μL of growth medium and treated with the compounds (1 μM), lipopolysaccharide (LPS; 1 μg/mL), or the corresponding vehicle (0.1% DMSO). Cell-free supernatants were collected after 18 h of incubation (37 °C, 5% CO_2_) and stored at −80 °C until tested. Cytokine production was determined with BD Cytometric Bead Array human inflammatory cytokines kit (BD Bioscience) on an Attune NxT flow cytometer (Thermo Fisher Scientific, Waltham, MA). Standard curves were generated using recombinant cytokines contained in the kit. The data were analyzed using the FlowJo (Tree Star, Inc., Ashland, OR) and Prism (GraphPad, San Diego, CA) software. Statistical significance was determined with one-way ANOVA with subsequent Bonferroni’s multiple comparisons test.

### RNA Sequencing

Peripheral blood mononuclear cells from three independent donors were seeded (2 × 10^6^ cells/mL) in 24-well plates in 1 mL of growth medium and treated with **4** (1 μM) or vehicle (0.1% DMSO) for 18 h at 37 °C in 5% CO_2_. The cells were washed with PBS, resuspended in RNAlater RNA stabilization solution (Sigma-Aldrich, St. Louis, MO), and stored at −80 °C. RNA extraction, library construction, and sequencing were conducted by Genewiz (Leipzig, Germany).

Briefly, total RNA was extracted using RNeasy mini kit (Qiagen, Hilden, Germany), according to the manufacturer’s protocol. RNA samples were quantified using a Qubit 4.0 fluorometer (Life Technologies, Carlsbad, CA), and RNA integrity was checked with RNA kit on a 5600 Fragment Analyzer (Agilent Technologies, Palo Alto, CA). All RNA samples were of high quality with an RNA quality number ≥9.4. RNA sequencing libraries were prepared using NEBNext Ultra II RNA library prep kit for Illumina, according to the manufacturer’s instructions (New England Biolabs, Ipswich, MA). Libraries were loaded on the Illumina NovaSeq. 6000 instrument and clustering was performed directly on the NovaSeq before sequencing according to the manufacturer’s instructions. The samples were sequenced using a 2 × 150 paired-end configuration. Image analysis and base calling were conducted by the NovaSeq Control Software. Raw sequence data (.bcl files) generated from the Illumina NovaSeq were converted into *fastq* files and de-multiplexed using the Illumina bcl2fastq 2.19 software. One mismatch was allowed for index sequence identification. After investigating the quality of the raw data, sequence reads were trimmed to remove possible adapter sequences and nucleotides with poor quality, using Trimmomatic v.0.36. The trimmed reads were mapped to the *Homo sapiens* reference genome, as available on ENSEMBL, using STAR aligner v.2.5.2b, thus generating BAM files. Unique gene hit counts were calculated using feature counts from the Subread package v.1.5.2. Only unique reads that fell within exon regions were counted. After extraction of gene hit counts, the gene hit counts table was used for downstream differential expression analysis.

Differential expression analysis was performed with iDEP.93.^62^ First, a low expression filter was applied (0.5 counts per million in at least 1 library). The remaining gene counts were normalized by counts per million in the EdgeR package, with a pseudo count of 4. Differential gene expression analysis was performed with the DESeq. 2 method, using a false discovery rate <0.05 and a gene expression fold-change >2 or <0.5 as the cutoff values. The list of differentially expressed genes was then used as input for gene annotation and pathway enrichment analysis with Metascape.^63^

### Peripheral Blood Mononuclear Cell Cytotoxicity

The PBMC cytotoxicity assays using K562 and MEC1 cells were performed as described previously, with some modifications.^64^ PBMCs were seeded (4 × 10^5^ cells/well) in duplicate in 96-well U-bottom plates and treated with the compounds (1 nM–1 μM) or vehicle (0.1% DMSO) for 18 h. K562 or MEC1 cells were stained with CFSE (Invitrogen, Carlsbad, CA), washed twice with complete medium, and added (1 × 10^4^ cells/well) to the pretreated PBMCs for a final effector cell to target tumor cell ratio of 40:1. After a 4 h coincubation (37 °C, 5% CO_2_), the cells were stained with Sytox blue dead cell stain (Invitrogen) and analyzed using an Attune NxT flow cytometer (Thermo Fisher Scientific, Waltham, MA) and the FlowJo software (Tree Star, Inc., Ashland, OR). Cells that were positive for both CFSE and Sytox blue were defined as dead K562 and MEC1 cells. The gating strategy is described in Figure S4. PBMCs alone and CFSE-labeled cancer cells alone were also treated with the compounds at the same concentrations and stained with Sytox blue to exclude any direct cytotoxicity of the compounds toward the PBMCs and cancer cells. Statistical significance was determined with one-way ANOVA with subsequent Bonferroni’s multiple comparisons test.

### NK Cell Degranulation, Activation, and Production of IFN-γ

PBMCs were seeded (5 × 10^5^ cells/well) in 96-well U-bottom plates and treated with the compounds (1 μM) or vehicle (0.1% DMSO) for 24 h. Anti-CD107a FITC and monensin (Biolegend, San Diego, CA) were added to all wells for the last 4 h of incubation. The cells were washed and stained with Live/Dead Fixable Aqua Dead Cell Stain (Invitrogen, Carlsbad, CA). After further washing, Fc receptors were blocked with Human TruStain FcX (Biolegend) and cells were stained for extracellular markers using anti-CD3 APC/Fire 750, anti-CD56 PE, and anti-CD69 PerCP-Cy5.5 antibodies (Biolegend). Intracellular staining was performed with an anti-IFN-γ APC antibody (Biolegend) after fixation and permeabilization using the Cyto-Fast Fix/Perm Buffer Set (Biolegend). Samples were analyzed using an Attune NxT flow cytometer (Thermo Fisher Scientific, Waltham, MA) and the FlowJo software (Tree Star, Inc., Ashland, OR). Following exclusion of dead cells, CD3^-^ CD56^+^ NK cells were evaluated for expression of CD107a, CD69, and IFN-γ. The gating strategy is described in detail in Figure S6. Statistical significance was determined with one-way ANOVA with subsequent Bonferroni’s multiple comparisons test.

### Bone-Marrow-Derived Dendritic Cell Antigen Presentation

CD4^+^ and CD8^+^ T cells were purified from splenocytes of OT II and OT I transgenic mice using CD4^+^ and CD8^+^ T-cell negative selection kits (Miltenyi Biotec, Germany), according to manufacturer instructions. Purified T cells were stained with CFSE (Invitrogen, Carlsbad, CA) and washed. Then, 5 × 10^4^ T cells were mixed in duplicate with 1 × 10^4^ BMDCs/well (pretreated with compounds [1 μM] or LPS [1 μg/mL] and 50 μg/mL ovalbumin (OVA) soluble protein [Invivogen, San Diego, CA] for 18 h, and then washed). After 72 h of coincubation (37 °C, 5% CO_2_), the supernatants were collected and stored at -80 °C for subsequent cytokine measurements. The cells were stained with Fixable viability dye eFluor 780 (eBioscience, Thermo Fisher Scientific, MA), anti-Thy1.2 PE-Cy7 (Biolegend, San Diego, CA), anti-CD8 eFluor450 (eBioscience), anti-CD4 eFluor450 (eBioscience), and anti-CD25 APC antibodies (Biolegend) and analyzed using a Beckman Coulter Cytoflex S flow cytometer (CA) and the FlowJo software (Tree Star, Inc., Ashland, OR). Live Thy1.2^+^/CD4^+^ and Thy1.2^+^/CD8^+^ were evaluated for CFSE dilution and CD25 expression. The gating strategy is described in detail in Figure S7.

Supernatants from T cells and BMDC cocultures were collected as described above. The cytokine concentrations were determined with Cytometric Bead Array Mouse Th1/Th2/Th17 cytokine kit (BD Bioscience) on an Attune NxT flow cytometer (Thermo Fisher Scientific). Standard curves were generated using recombinant cytokines contained in the kit. The data were analyzed using the FlowJo (Tree Star, Inc., Ashland, OR) and Prism (GraphPad, San Diego, CA) software. Statistical significance was determined with one-way ANOVA with subsequent Dunnett’s multiple comparisons test.

### Mouse Immunizations

Sex-matched NIH/OlaHsd mice (five per group) were immunized subcutaneously into the tail base with OVA (10 μg; Serva, Germany) alone or plus the compounds (50 μg) on days 0, 21, and 42. The injection volume in all of the experimental groups was 0.1 mL per mouse. On the seventh day after the second booster dose, the mice were anesthetized with i.p. application of ketamine/xylazine (25 mg/kg each) prior to blood collection from the axillary plexus. Individual sera from each animal were decomplemented at 56 °C for 35 min and then stored at −20 °C until tested.

### Measurement of Ovalbumin-Specific Serum Antibody Concentration

The levels of the OVA-specific total IgG, IgG1, and IgG2a in mice sera were determined by ELISA. Briefly, high-binding ELISA plates (Costar) were coated with a 15 μg/mL solution of OVA (Serva, Germany) in carbonate buffer (pH 9.6). Nonspecific antibody binding was blocked by 0.5% w/v bovine serum albumin in PBS-T (0.05% [v/v] Tween 20 in PBS) for 2 h at 37 °C. After washing, five serial dilutions of mice sera and standard preparations were added in duplicates. Plates were incubated overnight at room temperature, washed, and analyzed for OVA-specific IgG levels by incubation with HRP-conjugated goat anti-mouse IgG (2 h at 37 °C; Bio-Rad Laboratories), and then after washing, with 0.6 mg/mL o-phenylenediaminedihydrochloride solution in citrate-phosphate buffer, pH 5.0, with 0.5 μL 30% H_2_O_2_/mL for 30 min at room temperature in the dark. The enzymatic reaction was stopped with 12.5% H_2_SO_4_, and absorbance at 492 nm was measured using a microplate reader (Thermo Fisher Scientific, Waltham, MA).

For the determination of OVA-specific IgG1 and IgG2a, the plates were incubated with biotin-conjugated rat anti-mouse IgG1 or IgG2a (2 h at 37 °C; PharMingen, Becton Dickinson) and subsequently with streptavidin-peroxidase (Pharmingen) for 2 h at 37 °C. After washing, the substrate solution was added and incubated for 30 min at room temperature in the dark, as described above. The enzymatic reaction was stopped with 12.5% H_2_SO_4_ and absorbance at 492 nm was measured using a microplate reader. The relative quantities of antibodies were determined by parallel line assays using appropriate standard preparations of anti-OVA IgG, anti-OVA IgG1, and anti-OVA IgG2a antibodies with voluntarily assigned 20.000 AU/mL, 400.000 AU/mL, and 5000 AU/mL, respectively.^65^ Statistical significance was determined with one-way ANOVA with subsequent Dunnett’s multiple comparisons test.

### Screening against PAINS

All tested compounds were screened against the PAINS filter^66^ using the canvasSearch utility implemented in the Schrödinger software suite (Release 2021-2, New York). All compounds passed the PAINS filter.

### Statistics

Data analysis was performed using the Prism software (version 9; GraphPad Software, CA). Statistical differences were determined as specified in individual experimental procedures above. A *p* value <0.05 was considered statistically significant.

## Supporting Information Available

RNA-seq data (CSV)
SMILES_Strings (CSV)
^1^H and ^13^C NMR spectra and UHPLC traces of compounds **3** and **4** (PDF)

## Abbreviations Used

BMDC: bone-marrow-derived dendritic cell
CFSE: carboxyfluorescein succinimidyl ester
COMU: 1-[(1-(cyano-2-ethoxy-2-oxo-ethylideneaminooxy)-dimethylamino-morpholinomethylene)]methanaminium hexafluorophosphate
DIPEA: *N*,*N*-diisopropylethylamine
DMAP: 4-dimethylaminopyridine
EDC: 1-ethyl-3-(3-dimethylaminopropyl)carbodiimide
HOBt: hydroxybenzotriazole
KEGG: Kyoto Encyclopedia of Genes and Genomes
LPS: lipopolysaccharide
NF-κB: nuclear factor-κB
NK: natural killer
NOD2: nucleotide-binding oligomerization-domain-containing protein 2
OVA: ovalbumin
PAMP: pathogen-associated molecular pattern
PBMC: peripheral blood mononuclear cell
PRR: pattern recognition receptor
SEAP: secreted embryonic alkaline phosphatase
TFA: trifluoroacetic acid
TLR7: Toll-like receptor 7
