# Supplementary material for: Covalently Conjugated NOD2/TLR7 Agonists Are Potent and Versatile Immune Potentiators
Source: J Med Chem. 2022 Nov 6;65(22):15085–101. doi: 10.1021/acs.jmedchem.2c00808 (PMC9706565; doi:10.1021/acs.jmedchem.2c00808)
Supplement: Supplementary file 3 — jm2c00808_si_003.pdf [file jm2c00808_si_003.pdf]

# SUPPORTING INFORMATION

# Covalently conjugated NOD2/TLR7 agonists are potent and versatile immune potentiators

Samo Guzelj,[1] Matjaž Weiss,[1] Bram Slütter,[2] Ruža Frkanec,[3] and Žiga Jakopin*,[1]

[1]Faculty of Pharmacy, University of Ljubljana, SI-1000 Ljubljana, Slovenia

[2]Div. BioTherapeutics, Leiden Academic Centre for Drug Research, Leiden University, 2333 CC Leiden, The Netherlands

[3]Centre for Research and Knowledge Transfer in Biotechnology, University of Zagreb, 10000 Zagreb, Croatia

*Corresponding author:

Žiga Jakopin

Phone: +386 1 4769 646

Fax: + 386 1 4258 031

E-mail address: ziga.jakopin@ffa.uni-lj.si

## Table of contents:

1. Supporting Figures ........................................................................ S2

2. Supporting Tables ........................................................................ S8

3. NMR Spectra ........................................................................ S10

4. UHPLC traces ........................................................................ S12

## 1. Supporting Figures

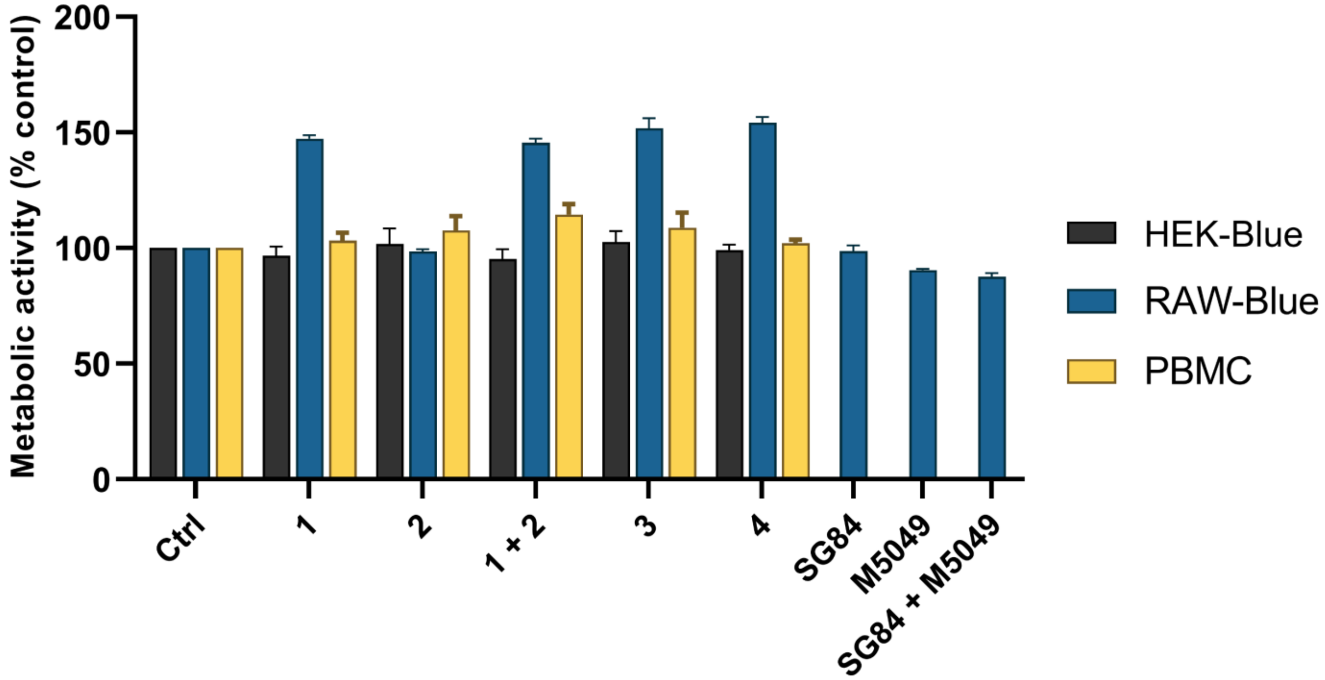


**Figure S1.** NOD2/TLR7 conjugates are not cytotoxic towards HEK-Blue and RAW-Blue cells, and PBMCs. Metabolic activities of HEK-Blue NOD2 and RAW-Blue cells, and PBMCs were measured after 18 h treatment with the compounds (10 µM), NOD2 antagonist **SG84** (5 µM) or TLR7 antagonist **M5049** (1 µM). Data are shown relative to the untreated control (0.1% DMSO). Data are means ±SEM of three (RAW-Blue cells) or four (HEK-Blue cells, PBMCs) independent experiments.

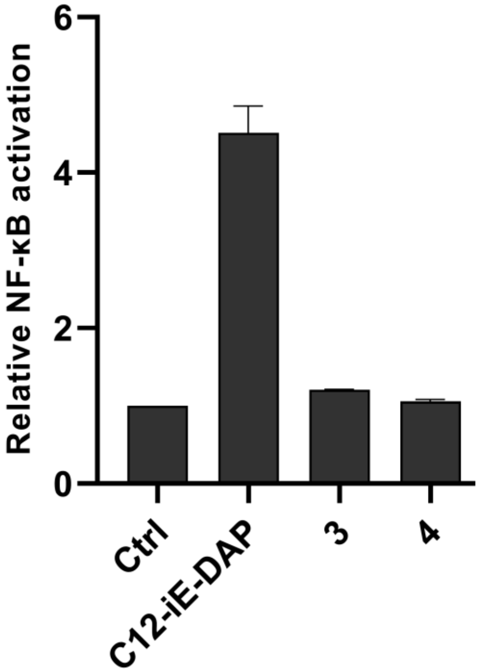


**Figure S2.** NOD2/TLR7 conjugates do not activate NOD1. HEK-Blue NOD1 cells were treated with C12-iE-DAP (100 nM) or the conjugates (10 µM) for 18 h. The activities are shown relative to the vehicle-treated control (0.1% DMSO). Data are means ±SEM of two independent experiments.

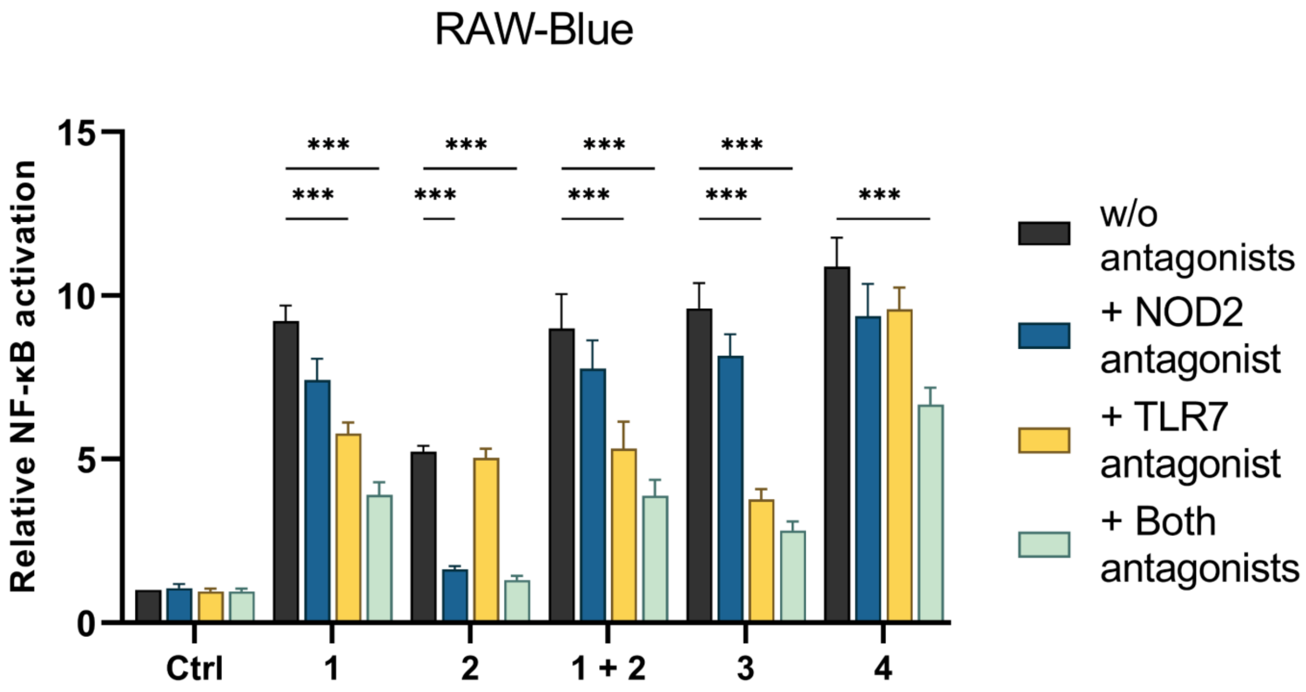


**Figure S3**. RAW-Blue cells were treated with the compounds (1 µM) for 18 h in the absence or presence of NOD2 antagonist **SG84** (5 µM), TLR7 antagonist **M5049** (1 µM), or their combination. The activities are shown relative to the vehicle-treated control (0.1% DMSO). Data are means ±SEM of three independent experiments. ***, p <0.001 *versus* relevant controls in the absence of antagonists.

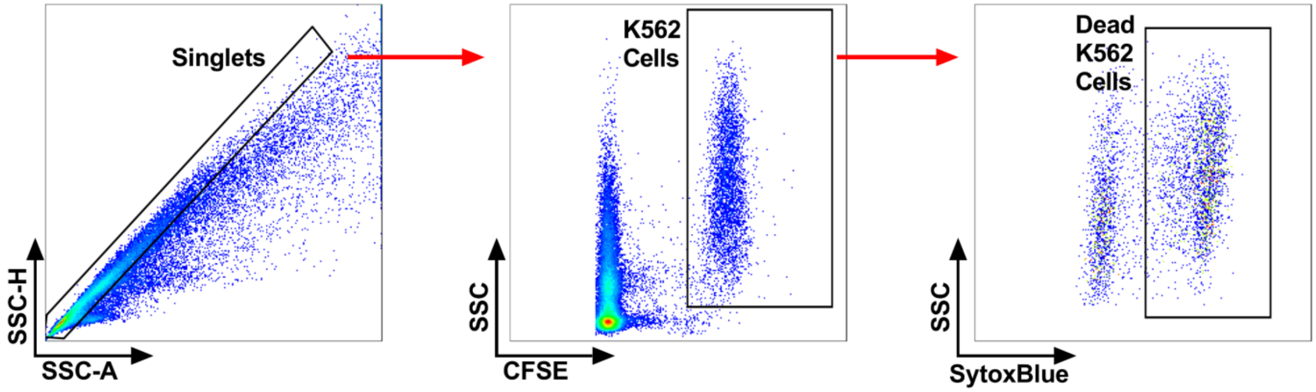


**Figure S4.** Representative gating strategy for the PBMC cytotoxicity assay. Singlets were first gated on a side scatter-A (SSC-A) vs. side scatter-H (SSC-H) plot. K562 cells were then identified as CFSE⁺. Finally, the ratio of dead K562 cells within the K562 population was determined by gating the SytoxBlue⁺ population.

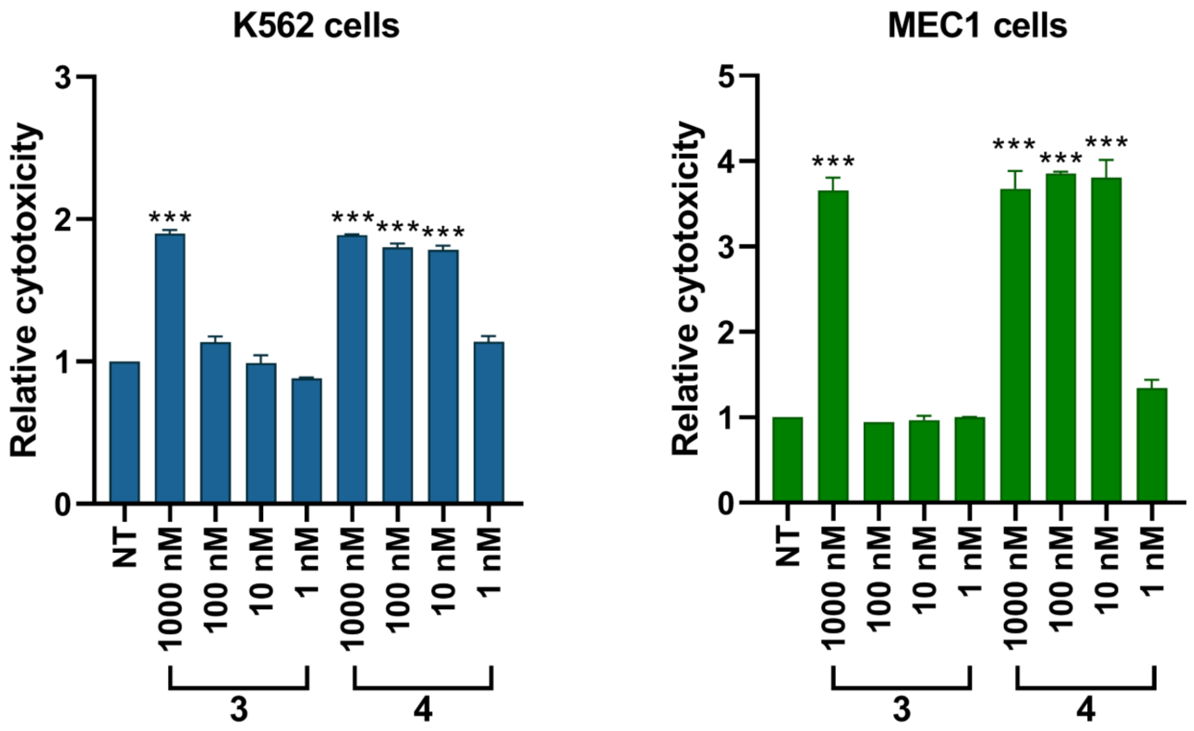


**Figure S5**. Concentration-dependent effects of conjugated NOD2/TLR7 diagonists on the induction of PBMC cytotoxicity. Following 18 h stimulation of PBMCs with compounds, CFSE labeled K562 or MEC1 cells were added. Cytotoxicity was determined after 4 h coincubation. Data are shown as relative activities to the vehicle-treated control (0.1% DMSO) and are means ±SEM of two independent experiments. ***, $p < 0.001$.

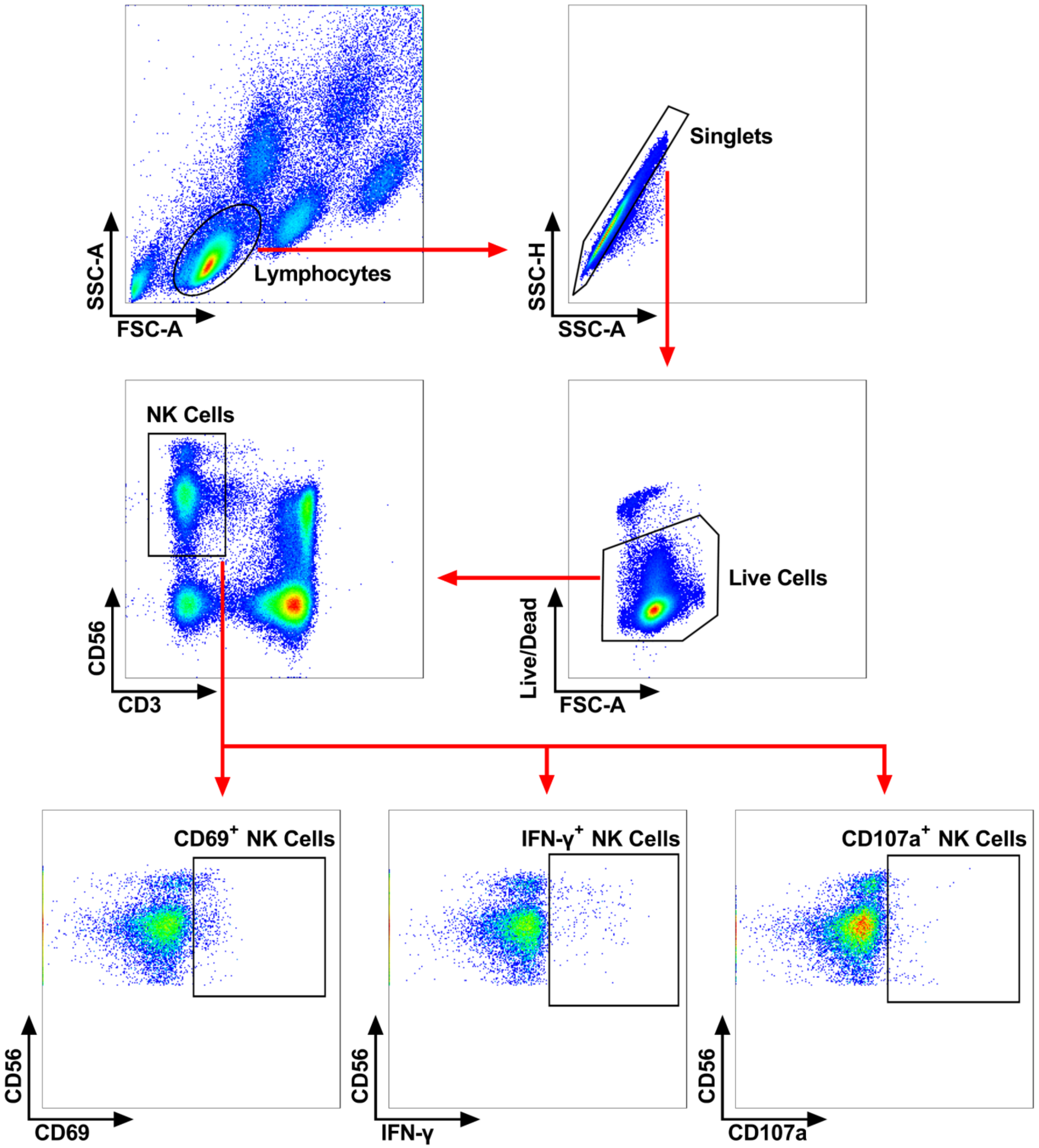


**Figure S6.** Representative gating strategy for the NK cell activation assay. First, lymphocytes were gated on a forward scatter-A (FSC-A) vs. side scatter-A (SSC-A) plot and single cells were gated on a side scatter-A (SSC-A) vs. side scatter-H (SSC-H) plot. Only viable cells were evaluated, identified by low intensity of the live/dead fixable aqua dead cell stain. Next, NK cells were identified as $CD3^-$, $CD56^+$. Finally, NK cell activation (CD69), degranulation (CD107a), and production of IFN-γ were evaluated within the NK cell population.

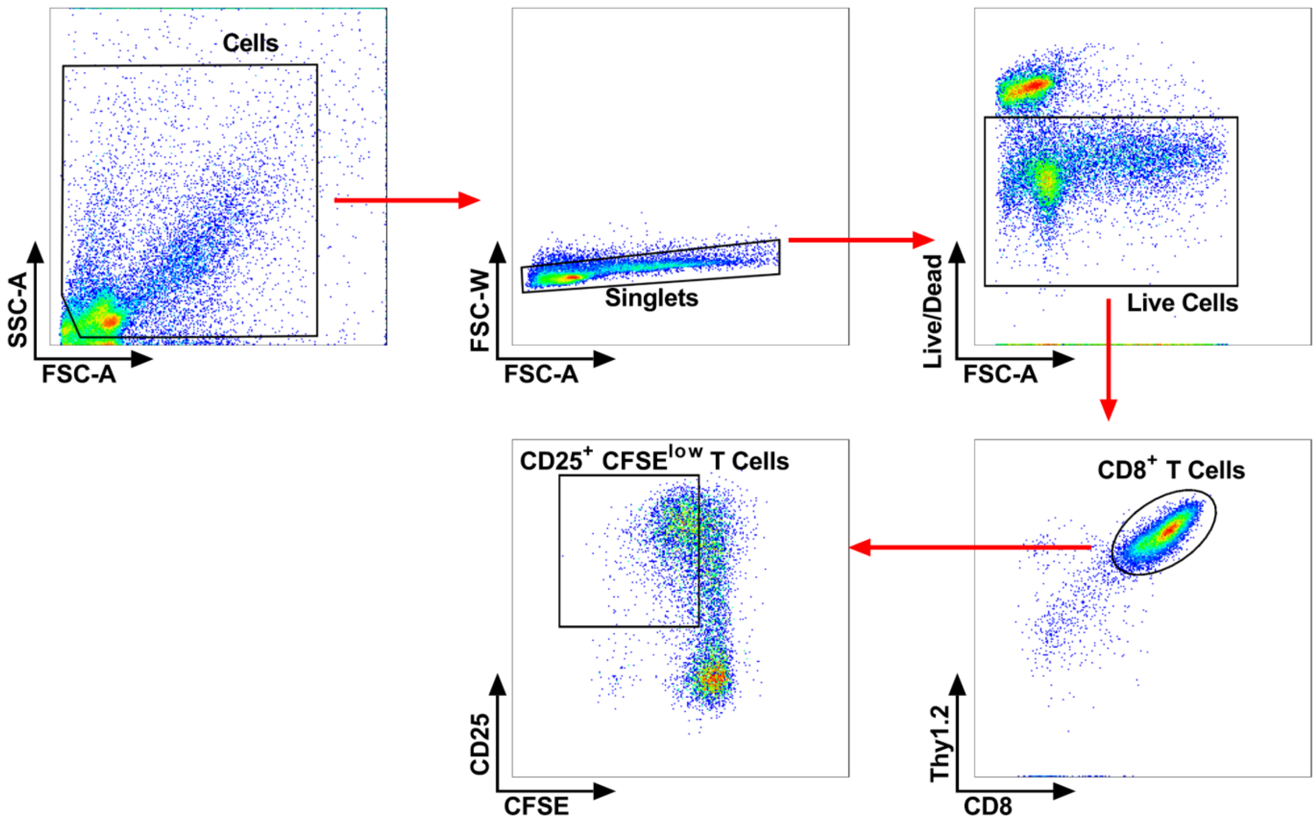


**Figure S7.** Representative gating strategy for the BMDC antigen presentation assay. First, cells were gated on a forward scatter-A (FSC-A) vs. side scatter-A (SSC-A) plot and single cells were gated on a side scatter-A (SSC-A) vs. side scatter-H (SSC-H) plot. Only viable cells were evaluated, identified by low intensity of the fixable viability dye. Next, $CD8^+$ T cells were identified as $CD8^+$, Thy1.$2^+$. Finally, T cell proliferation and activation were evaluated as CFSE dilution and CD25 expression, respectively. An analogous gating strategy was used for the experiment with $CD4^+$ T cells.

# 2. Supporting Tables

**Table S1.** Pathway enrichment analysis using the KEGG pathway database.

| Group | KEGG Pathway | Log (q-value) | Genes in KEGG pathway | | Enriched genes[a] |
|---|---|---|---|---|---|
| | | | Total | 4-enriched | |
| 1 | Cytokine-cytokine receptor interaction | -36.49 | 270 | 84 | **Up-regulated:** IL12B, CCL7, IFNG, CCL19, CCL8, IL6, CXCL1, CXCL13, IL17F, CXCL11, PRLR, CXCL8, LIFR, INHBA, TNFSF15, IL22, CXCL3, CXCL12, CCL3, CXCL2, MET, IL15RA, IL23R, TNFSF10, IL12RB2, CXCL6, CXCL9, CXCL10, CCL18, TNFRSF13B, XCR1, PDGFA, CCL4, FAS, IL26, IL2RA, IL18RAP, KITLG, TNFSF13B, IL19, FASLG, TNF, CCR7, CCL4L2, CD40, LTA, OSM, TNFSF9, CCL3L3, IL7, TNFSF4, CD70, IL10, CCL20, CSF1, IL18R1, IL15, CCR5, IFNLR1, FLT3, IL12A<br><br>**Down-regulated:** TGFBR1, IL13RA1, KIT, TNFSF11, CD27, TNFRSF11A, HGF, IL5RA, EDAR, TNFRSF21, TNFSF18, CX3CR1, CXCR1, CCL13, CXCR2, IL1R2, CCL16, CCL14, PDGFC, CSF1R, CCL24, KDR, CCR2 |
| 2 | NOD-like receptor signaling pathway | -13.88 | 170 | 42 | **Up-regulated:** IL6, CXCL1, OAS3, CXCL8, OAS1, CXCL3, CXCL2, OAS2, GBP5, IRF7, CARD17, STAT2, TXN, CASP12, GBP1, GBP4, AIM2, P2RX7, GBP7, TNF, MEFV, RBCK1, IFI16, RIPK2, GBP2, NFKBIA, GBP3, IRF9, NAMPT, TANK, NOD1, STAT1, CASP4, GSDMD<br><br>**Down-regulated:** MAPK13, PLCB2, NLRP12, NLRP3, CYBB, NLRC4, CARD9, CAMP |
| 3 | Hematopoietic cell lineage | -13.40 | 97 | 31 | **Up-regulated:** IL6, CD38, ITGB3, ITGA1, IL2RA, KITLG, CD22, TNF, IL7, MS4A1, CSF1, CD19, HLA-DOB, FLT3<br><br>**Down-regulated:** ITGA4, KIT, HLA-DMB, IL5RA, CD1D, GP1BA, CD4, CR1, CD33, ITGAM, IL1R2, CSF1R, CD9, CD36, CD14, CD1B, CD1E |
| 4 | Chemokine signaling pathway | -11.60 | 182 | 40 | **Up-regulated:** CCL7, CCL19, CCL8, CXCL1, CXCL13, CXCL11, CXCL8, CXCL3, CXCL12, CCL3, CXCL2, CXCL6, CXCL9, CXCL10, CCL18, STAT2, XCR1, CCL4, CCR7, CCL4L2, CCL3L3, NFKBIA, CCL20, STAT1, JAK2, CCR5, PTK2<br><br>**Down-regulated:** PLCB2, RASGRP2, ARRB1, PAK1, GNG7, CX3CR1, CXCR1, CCL13, CXCR2, CCL16, CCL14, CCL24, CCR2 |
| 5 | TNF signaling pathway | -9.00 | 115 | 28 | **Up-regulated:** IL6, CXCL1, CXCL3, CXCL2, CXCL10, FAS, EDN1, MMP14, MAP3K8, TNF, VCAM1, MLKL, LTA, NFKBIA, IRF1, CCL20, TANK, CSF1, IL18R1, PTGS2, IL15, CASP7, ICAM1<br><br>**Down-regulated:** MAPK13, MAP2K6, CREB5, MMP9, FOS |
| 6 | Pathways in cancer | -8.49 | 395 | 56 | **Up-regulated:** LAMA3, IL6, ARNT2, WNT5A, CCNA1, CXCL8, CXCL12, MET, PDGFA, RASGRP3, FAS, PML, TRAF4, LPAR3, KITLG, FASLG, PLD1, CDKN1A, PTGER4, NFKBIA, BRCA2, CDH1, CKS2, NFKB2, NKX3-1, STAT1, PTGS2, HIF1A, RALB, PTK2, FLT3<br><br>**Down-regulated:** ITGAV, AXIN2, DAPK2, TGFBR1, RASSF1, KIT, PLCB2, RASGRP2, GNAQ, RASGRP4, PTCH1, HGF, LAMA2, FZD1, FGF9, RXRA, GNG7, WNT10B, WNT7A, CEBPA, PPARG, CSF1R, FN1, MMP9, FOS |
| 7 | Rheumatoid arthritis | -8.38 | 99 | 25 | **Up-regulated:** IFNG, IL6, CXCL1, CXCL8, CTSL, CXCL12, CD80, CCL3, CXCL6, TNFSF13B, TNF, CCL3L3, CCL20, CSF1, IL15, ATP6V1H, HLA-DOB, ICAM1 |

| | | | | | |
|---|---|---|---|---|---|
| | | | | | **Down-regulated:** ATP6V0A1, TNFSF11, TNFRSF11A, HLA-DMB, ITGB2, FOS, ATP6V0D2 |
| 8 | Transcriptional misregulation in cancer | -8.31 | 204 | 37 | **Up-regulated:** IL6, ARNT2, CCNA1, CXCL8, ETV7, MET, PDGFA, PML, BCL2A1, GADD45B, NR4A3, CCR7, CD40, CDKN1A, GZMB, PAX5, PLAT, DDIT3, PTK2, AFF1, FLT3<br><br>**Down-regulated:** HPGD, LMO2, HHEX, SLC45A3, PBX1, RXRA, LDB2, ITGAM, DEFA3, IL1R2, CEBPA, PPARG, MPO, CSF1R, MMP9, CD14 |
| 9 | Phagosome | -8.28 | 168 | 33 | **Up-regulated:** CTSL, ITGB3, TUBA8, MSR1, TAP1, TUBB2A, TAP2, SCARB1, TUBA1C, ATP6V1H, HLA-DOB, HLA-A<br><br>**Down-regulated:** ITGAV, ATP6V0A1, DYNC2H1, FCGR3A, HLA-DMB, TLR6, ITGB2, CYBB, FCGR2B, ITGAM, CLEC7A, CD209, ITGB5, COLEC12, NOS1, MPO, CD36, CD14, OLR1, ATP6V0D2, MRC1 |
| 10 | NF-kappa B signaling pathway | -7.76 | 107 | 25 | **Up-regulated:** CCL19, CXCL8, CXCL12, CXCL2, DDX58, TRIM25, CCL4, BCL2A1, GADD45B, TNFSF13B, TNF, VCAM1, CCL4L2, CD40, LTA, NFKBIA, TANK, NFKB2, PTGS2, RELB, ICAM1<br><br>**Down-regulated:** TNFSF11, TNFRSF11A, CCL13, CD14 |
| 11 | Influenza A | -7.43 | 182 | 33 | **Up-regulated:** IL12B, IFNG, RSAD2, IL6, MX1, OAS3, CXCL8, OAS1, OAS2, TNFSF10, IRF7, EIF2AK2, IFIH1, DDX58, CXCL10, STAT2, TRIM25, FAS, PML, FASLG, TLR3, TNF, ADAR, NFKBIA, IRF9, STAT1, JAK2, HLA-DOB, IL12A, ICAM1<br><br>**Down-regulated:** MAPK13, HLA-DMB, NLRP3, MAP2K6 |
| 12 | Toll-like receptor signaling pathway | -7.36 | 104 | 24 | **Up-regulated:** IL12B,IL6, CXCL11, CXCL8, CD80, CCL3, IRF7, CXCL9, CXCL10, CCL4, MAP3K8, TLR3, TNF, CCL4L2, CD40, CCL3L3, NFKBIA, STAT1, IL12A<br><br>**Down-regulated:** MAPK13, TLR6, TLR5, MAP2K6, FOS, CD14 |

[a] Gene symbols are ordered by their $\log_2$(fold change) values after treatment with **4**, compared to the vehicle-treated (0.1% DMSO) control samples: highest to lowest.

**Table S2.** Experimentally determined solubilities of conjugates **3** and **4**.

| Compound | Solubility (µM) |
|---|---|
| **3** | 27.4 |
| **4** | 58.2 |

## 3. NMR Spectra

Compound **3**: $^{1}H$, 400 MHz, DMSO-$d_6$

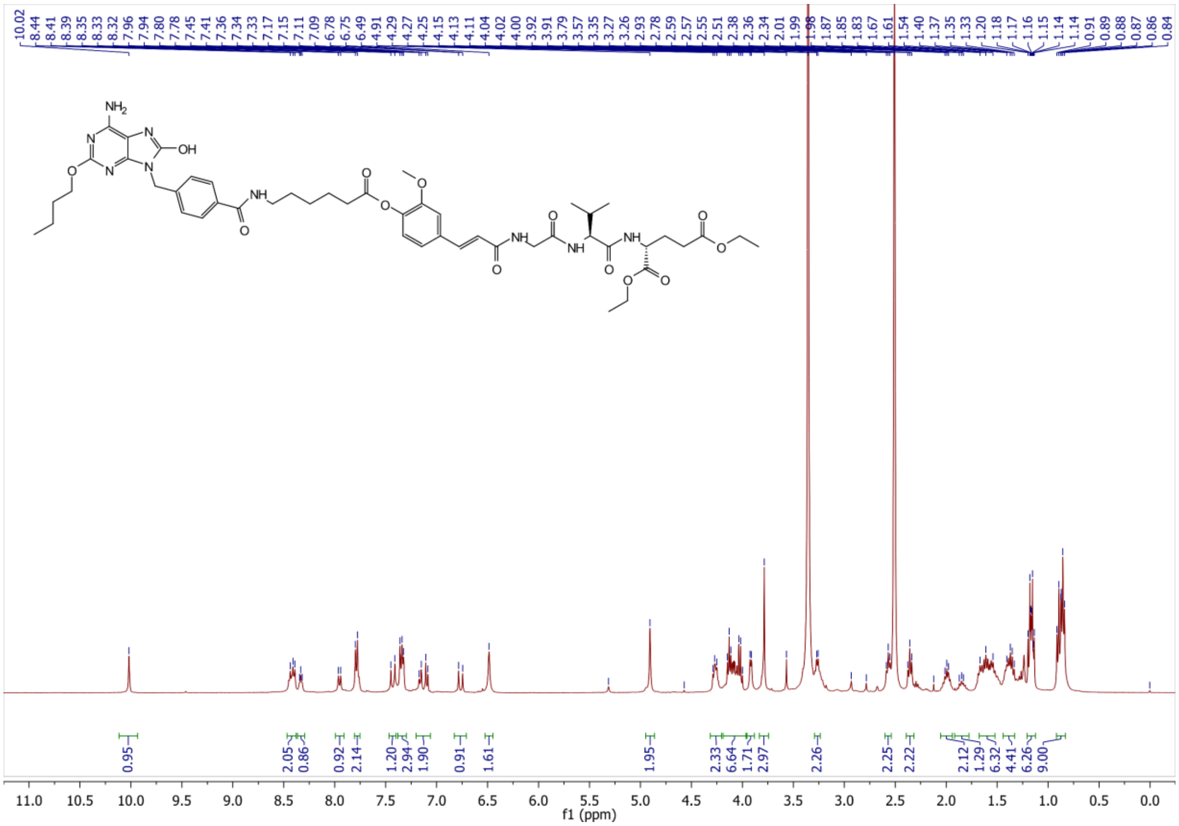

Compound **3**: $^{13}C$, 100 MHz, DMSO-$d_6$

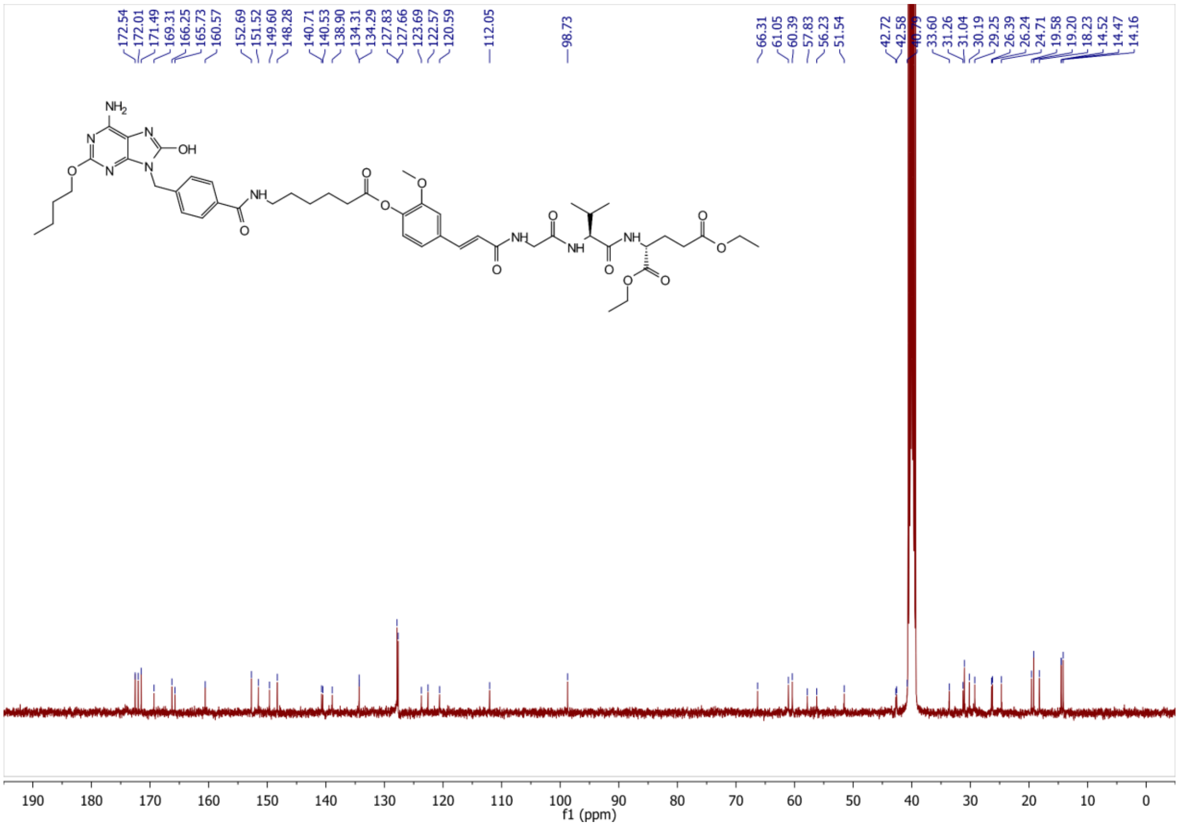

Compound **4**: $^{1}H$, 400 MHz, DMSO-$d_6$

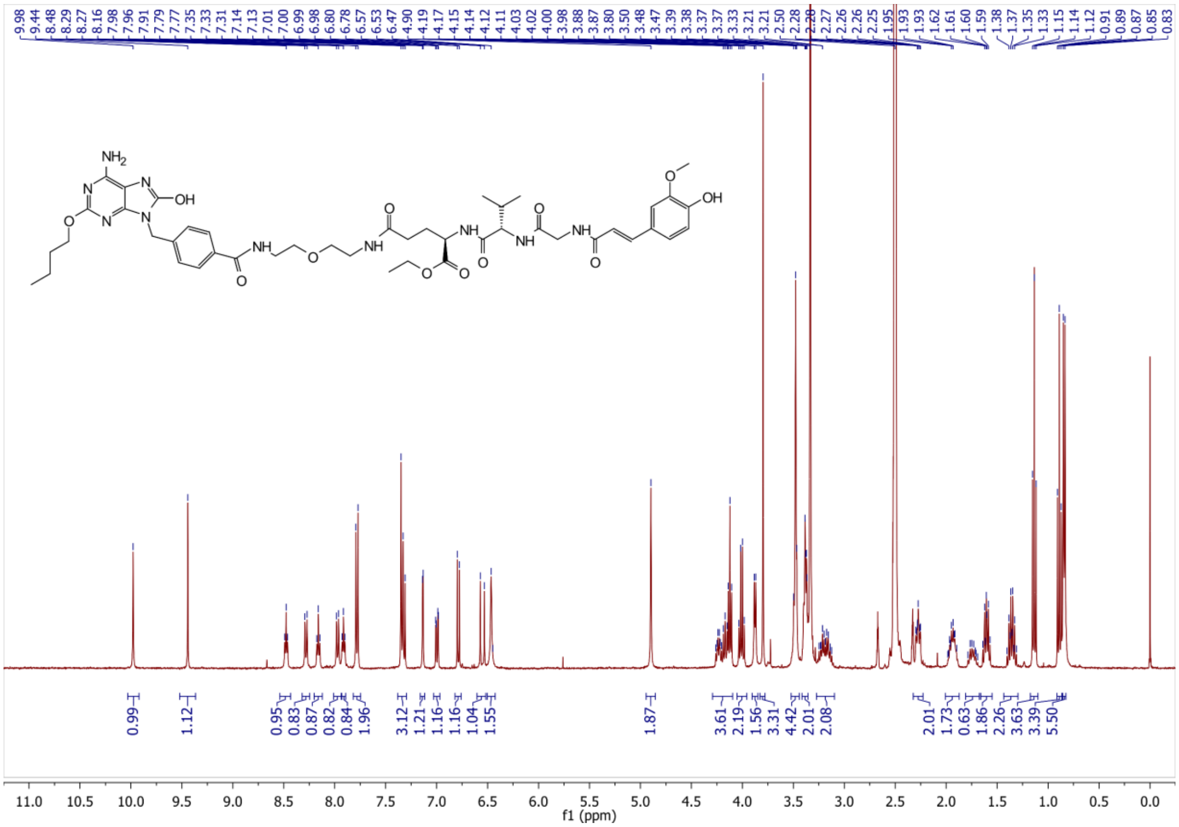

Compound **4**: $^{13}C$, 100 MHz, DMSO-$d_6$

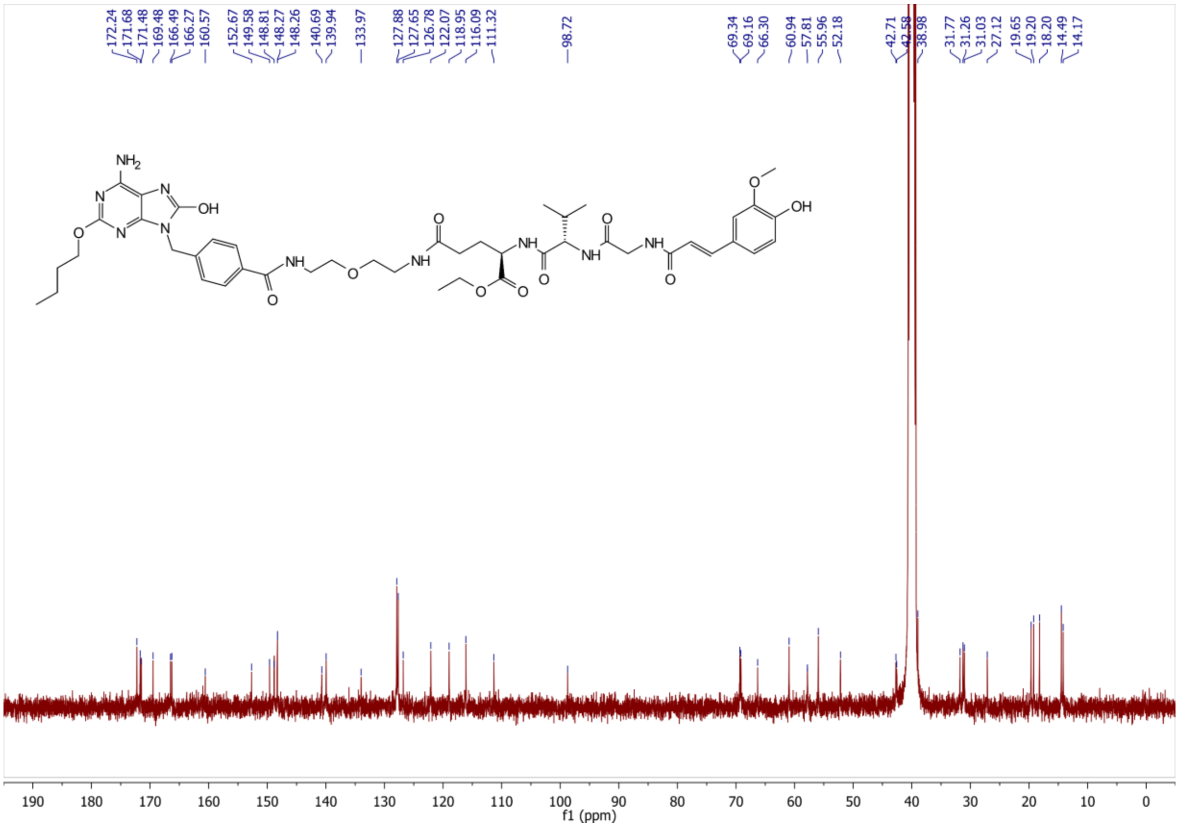

## 4. UHPLC traces

Compound **3**

# Chromatogram and Results

**Injection Details**

| | | | |
|---|---|---|---|
| *Injection Name:* | **Conjugate 3** | *Run Time (min):* | **12,50** |
| *Processing Method:* | **Quantitative** | *Wavelength:* | **254** |

**Chromatogram**

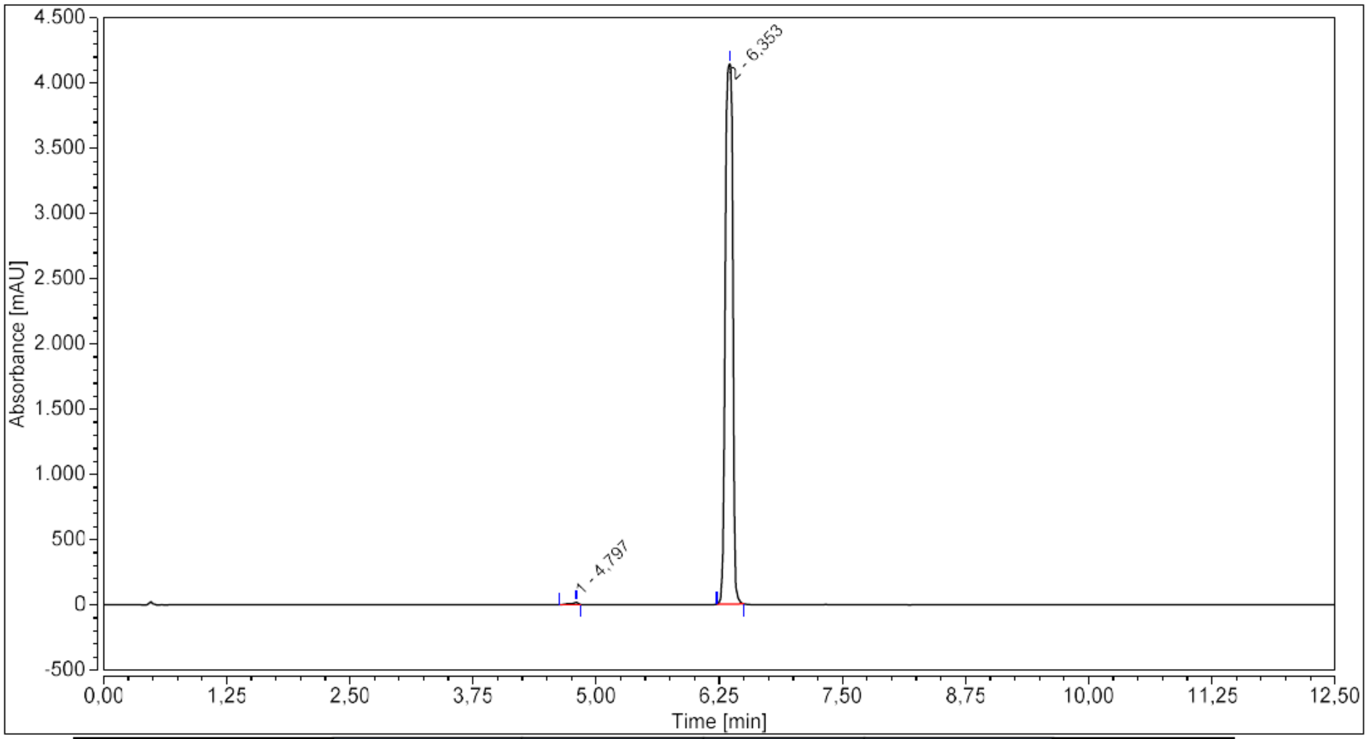


**Integration Results**

| No. | Retention Time min | Area mAU*min | Height mAU | Relative Area % | Relative Height % |
|---|---|---|---|---|---|
| 1 | 4,797 | 1,532 | 18,120 | 0,42 | 0,44 |
| 2 | 6,353 | 365,051 | 4142,543 | 99,58 | 99,56 |
| **Total:** | | **366,583** | **4160,663** | **100,00** | **100,00** |

Compound **4**

## Chromatogram and Results

### Injection Details

| | | | |
|---|---|---|---|
| *Injection Name:* | **Conjugate 4** | *Run Time (min):* | **12,50** |
| *Processing Method:* | **Quantitative** | *Wavelength:* | **254** |

### Chromatogram

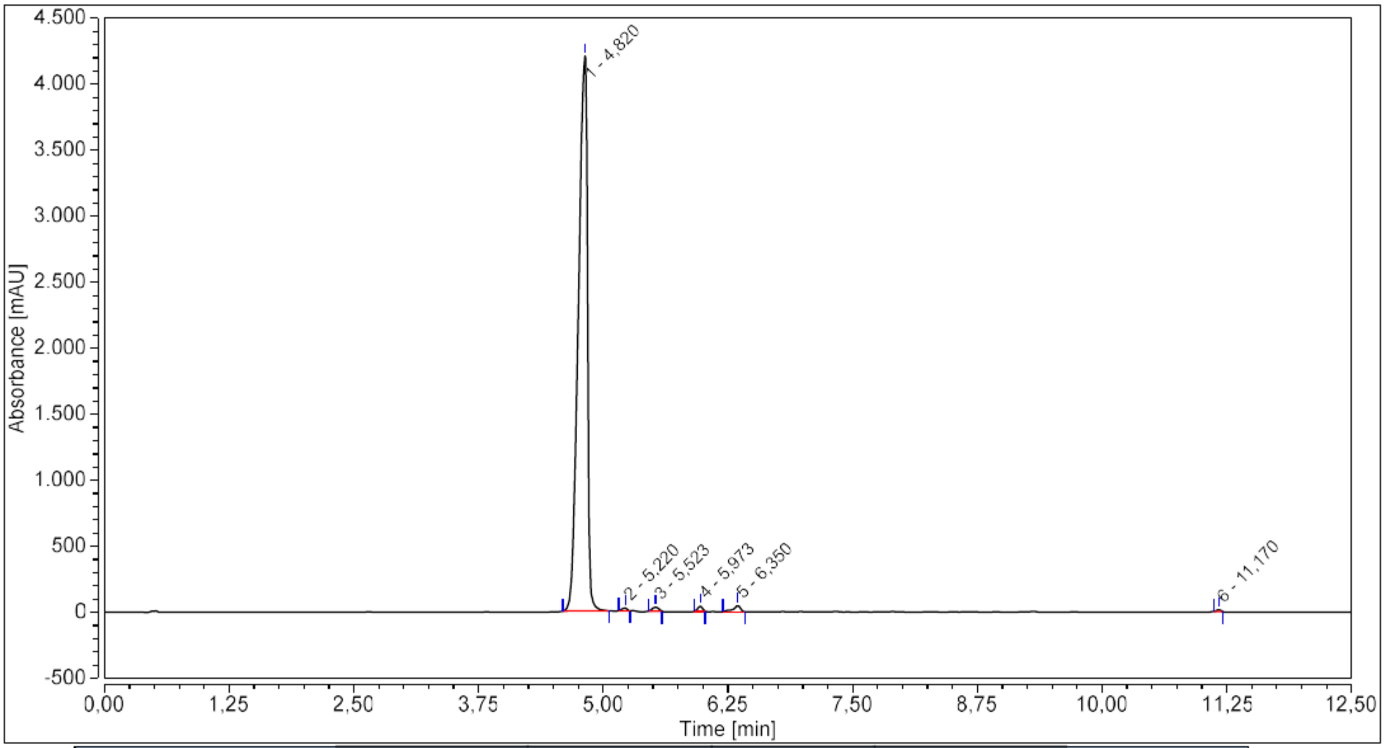


### Integration Results

| No. | Retention Time min | Area mAU*min | Height mAU | Relative Area % | Relative Height % |
|---|---|---|---|---|---|
| 1 | 4,820 | 446,231 | 4203,276 | 97,79 | 96,43 |
| 2 | 5,220 | 1,409 | 22,839 | 0,31 | 0,52 |
| 3 | 5,523 | 2,251 | 31,352 | 0,49 | 0,72 |
| 4 | 5,973 | 2,043 | 39,558 | 0,45 | 0,91 |
| 5 | 6,350 | 3,719 | 46,260 | 0,82 | 1,06 |
| 6 | 11,170 | 0,684 | 15,404 | 0,15 | 0,35 |
| **Total:** | | **456,338** | **4358,690** | **100,00** | **100,00** |
